# Supplementary material for: Factors associated with vaccine coverage improvements in Senegal between 2005 and 2019: a quantitative retrospective analysis
Source: BMJ Open. 2023 Oct 4;13(10):e074388. doi: 10.1136/bmjopen-2023-074388 (PMC10551957; doi:10.1136/bmjopen-2023-074388)
Supplement: Supplementary data [file bmjopen-2023-074388supp001.pdf]

REGION	2005	2010/2011	2012/2013	2014	2015	2016	2017	2018	2019
Dakar	150	141	70	130	52	63	164	80	60
Diourbel	229	206	139	259	113	107	182	110	107
Fatick	178	185	77	163	94	97	184	90	63
Kaffrine	*	188	118	245	116	140	217	161	106
Kaolack	255	217	131	259	146	124	156	119	130
Kedougou	*	87	66	134	78	100	142	76	60
Kolda	232	190	108	192	132	104	164	94	93
Louga	198	165	82	199	97	86	158	92	77
Matam	178	160	92	182	87	96	180	84	94
Saint-Louis	169	157	87	179	59	93	137	79	67
Sedhiou	*	197	95	175	83	78	196	92	85
Tambacounda	183	178	99	222	91	103	201	106	91
Thies	232	179	118	214	111	95	190	98	107
Ziguinchor	134	127	47	109	51	37	119	56	43
TOTAL	2138	2377	1329	2662	1310	1323	2390	1337	1183

*** *Kaffrine, Kedougou, and Sedhiou were split from existing regions in 2008.***

Table S1 Unweighted number of survey respondents fitting survey criteria (living children ages 12-24 months) by year and region.

Region	DTP3	Female Literacy	Skilled Prenatal Care	Access to Care Concerns				Urban
				Traveling Alone	*Distance*	*Permission*	*Money*	
**Dakar**	95.2%	45.2%	100.0%	12.1%	14.8%	2.9%	36.0%	95.2%
**Diourbel**	93.2%	14.3%	99.3%	2.8%	30.0%	9.2%	52.7%	9.7%
**Fatick**	91.1%	33.2%	96.4%	12.4%	35.2%	8.9%	45.4%	24.8%
**Kaffrine**	94.0%	13.1%	98.2%	19.2%	21.4%	25.5%	46.0%	13.8%
**Kaolack**	93.5%	24.8%	98.4%	3.7%	34.7%	1.3%	53.4%	31.2%
**Kedougou**	83.3%	24.6%	89.6%	21.0%	7.0%	19.9%	32.3%	20.2%
**Kolda**	73.3%	23.7%	93.5%	45.2%	44.5%	2.0%	61.3%	20.7%
**Louga**	84.0%	26.3%	95.9%	26.5%	40.8%	18.6%	60.4%	17.9%
**Matam**	100.0%	10.3%	97.4%	20.5%	30.9%	21.3%	84.3%	13.8%
**Saint-Louis**	94.2%	27.8%	97.0%	13.8%	40.0%	20.4%	70.5%	38.8%
**Sedhiou**	88.3%	28.5%	95.4%	50.2%	60.2%	2.4%	66.7%	12.6%
**Tambacounda**	85.5%	10.3%	91.8%	21.8%	2.9%	20.9%	39.3%	34.7%
**Thies**	96.6%	30.0%	100.0%	16.3%	27.7%	4.9%	45.7%	47.4%
**Ziguinchor**	97.7%	61.9%	97.2%	14.1%	16.6%	2.3%	43.6%	40.1%
**All Senegal**	92.1%	26.9%	97.8%	15.9%	28.2%	9.5%	50.9%	37.9%

Table S2 Weighted percentage of survey respondents with "yes" responses per household factor for year 2019. Data is filtered for respondents who meet inclusion criteria.

Variable	β Coefficients and 95% CI	p-value
Urban	0·16 (-0·36, 0·72)	0·547
**Female Literacy**	**0·92 (0·30, 1·63)**	**0·0065**
**Skilled Prenatal Care from a doctor, nurse, or midwife**	**1·58 (0·82, 2·30)**	**< 0·0001**
Problems experienced among respondents who sought care when sick: Women ages 15-49 were asked if the following were significant barriers when seeking treatment when they were sick.		
Access to Care - Distance	-0·23 (-0·77, 0·32)	0·400
Access to Care - Permission	0·26 (-0·39, 0·98)	0·463
**Access to Care - Concerns over Traveling Alone**	**-0·82 (-1·35, -0·28)**	**0·0026**
Access to Care - Money for Care	0·02 (-0·45, 0·50)	0·919

Table S3 Household-level factors associated with DTP3 vaccination by survey response per living child age 12-23 months (N=1129 (weighted)): multivariate regression analysis. DHS 2019.

Smalley HK, *et al. BMJ Open* 2023; 13:e074388. doi: 10.1136/bmjopen-2023-074388

Variable	Coefficients and 95% CI	p-value
Poverty Incidence	-0·249 (-0·58, 0·08)	0·126
Doctors per 100,000 population	-0·352 (-1·72, 1·01)	0·579
Nurses and Midwives per 100,000 population	0·323 (-0·16, 0·81)	0·170

Table S4 Region-level factor associations with DTP3 coverage at the regional level (N=14): multivariate linear regression analysis. DHS 2019.

Smalley HK, *et al. BMJ Open* 2023; 13:e074388. doi: 10.1136/bmjopen-2023-074388

**Sociodemographic Characteristics of Survey Respondents (n=8,649)**	
**Religion**	
Muslim	97%
Christian	3%
**Ethnicity**	
Wolof	40%
Poular	28%
Serer	15%
Mandingue/Soce	5%
Diola	4%
Soninke	3%
Other/non-Senegalese	5%
**Education**	
Primary	21%
Secondary or more	32%
**Marital Status**	
Single	30%
Married	65%
Divorced/separated	4%
Widow	1%
**Age**	
15-19	22%
20-24	19%
25-29	16%
30-34	15%
35-39	13%
40-44	8%
45-49	7%

Table S5 Sociodemographic characteristics of female survey respondents, 2019.
