# Supplementary material for: Factors associated with vaccine coverage improvements in Senegal between 2005 and 2019: a quantitative retrospective analysis
Source: BMJ Open. 2023 Oct 4;13(10):e074388. doi: 10.1136/bmjopen-2023-074388 (PMC10551957; doi:10.1136/bmjopen-2023-074388)
Supplement: Author's manuscript [file bmjopen-2023-074388.draft_revisions.pdf]

BMJ Open is committed to open peer review. As part of this commitment we make the peer review history of every article we publish publicly available.

When an article is published we post the peer reviewers' comments and the authors' responses online. We also post the versions of the paper that were used during peer review. These are the versions that the peer review comments apply to.

The versions of the paper that follow are the versions that were submitted during the peer review process. They are not the versions of record or the final published versions. They should not be cited or distributed as the published version of this manuscript.

BMJ Open is an open access journal and the full, final, typeset and author-corrected version of record of the manuscript is available on our site with no access controls, subscription charges or pay-per-view fees (http://bmjopen.bmj.com).

If you have any questions on BMJ Open's open peer review process please email info.bmjopen@bmj.com

BMJ Open

Factors associated with vaccine coverage improvements in Senegal

Journal:	*BMJ Open*
Manuscript ID	bmjopen-2023-074388
Article Type:	Original research
Date Submitted by the Author:	05-Apr-2023
Complete List of Authors:	Smalley, Hannah; Georgia Institute of Technology, H. Milton Stewart School of Industrial and Systems Engineering<br>Castillo-Zunino, Francisco; Amazon, Amazon Modeling and Optimization<br>Keskinocak, Pinar; Georgia Institute of Technology, ISYE<br>Nazzal, Dima; Georgia Institute of Technology, Industrial and Systems Engineering<br>Sakas, Zoë; Emory University, Gangarosa Department of Environmental Health, Rollins School of Public Health<br>Sarr, Moussa; Institut de Recherche en Santé de Surveillance Epidemiologique et de Formation<br>Freeman, Matthew; Emory Univ, Gangarosa Department of Environmental Health
Keywords:	Health policy < HEALTH SERVICES ADMINISTRATION & MANAGEMENT, Public health < INFECTIOUS DISEASES, Paediatric infectious disease & immunisation < PAEDIATRICS

SCHOLARONE™ Manuscripts

I, the Submitting Author has the right to grant and does grant on behalf of all authors of the Work (as defined in the below author licence), an exclusive licence and/or a non-exclusive licence for contributions from authors who are: i) UK Crown employees; ii) where BMJ has agreed a CC-BY licence shall apply, and/or iii) in accordance with the terms applicable for US Federal Government officers or employees acting as part of their official duties; on a worldwide, perpetual, irrevocable, royalty-free basis to BMJ Publishing Group Ltd ("BMJ") its licensees and where the relevant Journal is co-owned by BMJ to the co-owners of the Journal, to publish the Work in this journal and any other BMJ products and to exploit all rights, as set out in our licence.

The Submitting Author accepts and understands that any supply made under these terms is made by BMJ to the Submitting Author unless you are acting as an employee on behalf of your employer or a postgraduate student of an affiliated institution which is paying any applicable article publishing charge ("APC") for Open Access articles. Where the Submitting Author wishes to make the Work available on an Open Access basis (and intends to pay the relevant APC), the terms of reuse of such Open Access shall be governed by a Creative Commons licence – details of these licences and which Creative Commons licence will apply to this Work are set out in our licence referred to above.

Other than as permitted in any relevant BMJ Author's Self Archiving Policies, I confirm this Work has not been accepted for publication elsewhere, is not being considered for publication elsewhere and does not duplicate material already published. I confirm all authors consent to publication of this Work and authorise the granting of this licence.

For peer review only

Factors associated with vaccine coverage improvements in Senegal

Hannah K Smalley[1], Francisco Castillo-Zunino[2i], Pinar Keskinocak,[1,3], Dima Nazzal[1], Zoe Sakas[4], Moussa Sarr[5], Matthew C. Freeman[4]*

1 H. Milton Stewart School of Industrial and Systems Engineering, Georgia Institute of Technology, 755 Ferst Dr., NW, Atlanta, GA, USA.

2 Amazon Modeling and Optimization, Amazon, Bellevue, WA, USA.

3 Center for Health and Humanitarian Systems, Georgia Institute of Technology, 755 Ferst Dr., NW, Atlanta, GA, USA.

4 Gangarosa Department of Environmental Health, Rollins School of Public Health, Emory University, Atlanta, GA, USA

5 Institut de Recherche en Santé de Surveillance Epidemiologique et de Formation, Dakar, Senegal

* Corresponding author's email address: hannahsmalley@gatech.edu

[i] Work performed while the author was with Georgia Institute of Technology prior to joining Amazon.

For peer review only - http://bmjopen.bmj.com/site/about/guidelines.xhtml

ABSTRACT

Objective Senegal has demonstrated catalytic improvements in national coverage rates for early childhood vaccination, despite lower development assistance for childhood vaccines in Senegal compared to other low and lower-middle income countries. Understanding factors associated with historical changes in childhood vaccine coverage in Senegal, as well as heterogeneities across its 14 regions, can highlight effective practices that might be adapted to improve vaccine coverage elsewhere.

Design Childhood vaccination coverage rates, demographic information, and health system characteristics were identified from Senegal's Demographic and Health Surveys (DHS) and Senegal national reports for years 2005 to 2019. Multivariate logistic and linear regression analyses were performed to determine statistical associations of demographic and health system characteristics with respect to childhood vaccination coverage rates.

Setting The 14 administrative regions of Senegal were chosen for analysis.

Participants DHS survey respondents corresponding to a total of 1183 living children aged 12-23 months were considered for household-level analysis.

Outcome Measures Immunization with the third dose of the diphtheria-tetanus-pertussis vaccine (DTP3), widely used as a proxy for estimating immunization coverage levels and the retention of children in the vaccine program.

Results Factors associated with childhood vaccination coverage include urban residence, female literacy, skilled prenatal care, and self-reported ease of access to care when sick, considering travel distance to a healthcare facility and concerns over traveling alone. Higher coverage with less variability over time was reported in urban regions near the capital and the coast, with increased coverage in recent years in more rural and landlocked regions.

For peer review only

Conclusions Childhood vaccination was more likely among children whose mothers had higher literacy, received skilled prenatal care, and had perceived ease of access to care when sick. Overall, vaccination coverage is high in Senegal and disparities in coverage between regions have decreased significantly in recent years.

Strengths and limitations of the study

- This study's main strength was to be the first to explain associations between DTP3 coverage at the subnational level within Senegal. DTP3 is a widely used proxy for estimating immunization coverage levels and the retention of children in the vaccine program. Senegal is a lower-middle income country which has experienced success in increasing vaccination coverage at the national level in comparison to other comparable low and lower-middle income countries.
- This study generated evidence about changes in vaccination coverage over time by geographic region.
- Analysis was not performed at the district level and thus heterogeneities at the subregional level were not evaluated.

Keywords: Childhood immunization coverage; Vaccines; Senegal; Inequality; Regression analysis

For peer review only - http://bmjopen.bmj.com/site/about/guidelines.xhtml

Background

Vaccination is one of the most cost-effective and influential public health interventions [1]. For each dollar spent on routine and supplementary vaccinations in low- and middle-income countries from 2011-2020, the average country-level return on investment is estimated to be 44 times the cost [2]. High vaccination coverage is necessary for controlling, eliminating, or eradicating vaccine-preventable diseases [3]. Gavi, The Vaccine Alliance (Gavi), was launched in 2000 and is one of the main sources of vaccine financing for eligible low- and middle-income countries. Some but not all Gavi-supported countries were able to reach the 2015 Global Vaccine Action Plan (GVAP) goal of 90% coverage for the third dose of the diphtheria-tetanus-pertussis vaccine (DTP3) by 2010 [4, 5].

In 2017, Africa reported a DTP3 coverage of 72%, the lowest percentage of all the World Health Organization (WHO) regions [4]. Within the African Region, several countries have outperformed their peers with regards to high and sustained vaccination coverage. DTP3 coverage in Senegal increased from 52% to 93% [6], outperforming other low income (LIC) and lower-middle income (LMIC) countries, despite lower development assistance for health (DAH) relative to many other countries in this group (more details provided in the supplemental materials). The Government of Senegal has demonstrated a commitment to improving access to health care, including childhood vaccination, which likely contributed to the country's success [7-9]. However, like other countries in sub-Saharan Africa, Senegal's childhood vaccine coverage was not consistent sub-nationally.

The purpose of this study was to identify factors associated with DTP3 coverage in Senegal at both the household level and regionally. DTP3 is often used as a proxy for estimating the retention of children in the vaccine program [3]. Subnational trends in early childhood vaccine coverage often illustrate inequities in vaccine service delivery [10, 11], and looking at national-level data alone may overestimate vaccine system performance [12]. Factors statistically associated with full childhood immunization coverage have been identified in previous studies [13-16]. We complement prior work by focusing on DTP3 coverage in Senegal, with an analysis of changes in coverage over time by region from 2005-2019. We analyzed

For peer review only - http://bmjopen.bmj.com/site/about/guidelines.xhtml

For peer review only

geographic and population factors, including female literacy and access to skilled prenatal care. We also considered factors aggregated at the regional level including poverty and the number of healthcare workers.

Methods

We conducted a study to assess the demographic associations of early childhood vaccination within Senegal, including assessing the impact of heterogeneities at the sub-national level. For the study period of 2005-2019, we analyzed geographic and population factors for each of Senegal's 14 regions to examine potential associations with DTP3 coverage. The analysis was conducted as part of the Vaccine Exemplars Program, the purpose of which was to identify how and why some countries have achieved better-than-average and sustained coverage of early childhood vaccines [9, 17-19].

Data sources

Household-level immunization data was collected from Senegal's Demographic and Health Surveys (DHS) (years 2005, 2010/2011, 2012/2013, and yearly from 2014 to 2019) [20], with data provided for all 14 regions and all years (notably 3 of the regions were created/split from existing regions in 2008; coverage for these regions in 2005 is estimated to be equal to that of the original combined regions). DHS surveys were sampled to be representative at the regional level, and thus we were not able to analyze statistical associations at the smaller district level. Data extracted from the surveys include DTP3 vaccination (recommended administration to children at 14-weeks-old in Senegal), residence type (urban or rural), female literacy, proportion receiving prenatal care from skilled providers, and self-reported problems accessing care when sick (i.e., women were asked if distance, permission, money, and traveling alone were significant barriers when seeking treatment). Region-specific population density and poverty (reported for year 2018) were determined from Senegal national reports [21]. We collected regional counts of skilled healthcare workers from the Ministère de la Santé et de l'Action sociale (MSAS) (2018) [22]. Data sources and corresponding years are summarized in Table 1.

For peer review only - http://bmjopen.bmj.com/site/about/guidelines.xhtml

Data	Source	Years
Senegal household survey data including: • Vaccinations<br>• Urban/rural residence<br>• Female literacy<br>• Self-reported problems accessing care when sick<br>• Prenatal care provider	DHS [20]	2005, 2010/2011, 2012/2013, and yearly from 2014-2019
Regional population density, poverty (country specific metric)	Senegal national reports via Knoema.com [21]	2018
Regional doctors, nurses, midwives (counts)	MSAS [22]	2018

Table 1 Data sources and years.

Data analysis

Bivariate and multivariate logistic and linear regression analyses were performed to determine statistical associations of factors with respect to coverage of DTP3. We calculated DTP3 coverage – the dependent variable in our models - for each region of Senegal using DHS data for each year available during the study period. The immunization coverage for a region was calculated as the percentage of living children ages 12-23 months represented in the survey for that region who received DTP3. We calculated the Pearson correlation coefficients (r) to determine statistically significant correlations between the quantitative factors and between factors and DTP3 coverage. We clustered geographic regions by location and performed independent sample t-tests to compare the average change in DTP3 coverage over time.

For peer review only

No patient or public involvement

There was no patient or public involvement in the study design of this research, the interpretation of the results, or the writing or editing of this document.

Results

Factors associated with DTP3 coverage by region

DTP3 coverage by region ranged from 73% to 100% in 2019 (Figure 1). Higher DTP3 coverage rates were observed in Dakar (the most populous region and includes the capital city) and its surrounding regions, and along the coast, as well as in the landlocked region of Matam. In 2018, there were approximately 6,600 people per km^2 in Dakar, which is predominantly urban (97% urban) [23]. The population density in other regions was at most 362 people per km^2. Population density does not have a statistically significant correlation with DTP3 coverage at the regional level, though this correlation is higher when excluding Dakar (Pearson's correlation r=0·39, p=0·19).

Our unadjusted bivariate analyses (Figure 2) revealed significant associations (p=0·05) between higher DTP3 vaccination and the following:

- positive associations:
    - type of place of residence (urban) (β=0·61, p=0·0157),
    - higher female literacy (β=1·11, p=0·0007),
    - higher skilled prenatal care (β=1·80, p<0·0001), and
- negative associations:
    - more barriers when seeking treatment with respect to
        - distance (β=-0·70, p=0·0009) and

For peer review only - http://bmjopen.bmj.com/site/about/guidelines.xhtml

139 ▪ traveling alone (β=-1·08, p<0·0001).

140 Multivariate regression revealed correlations between factors (Figure 2), with only 3 factors remaining
141 statistically significant: positive associations with female literacy (β=0·92, p=0·0065) and skilled prenatal
142 care (β=1·58, p<0·0001), and negative associations with more barriers when seeking treatment with
143 respect to traveling alone (β=-0·82, p=0·0026) (more details provided in the supplemental materials). We
144 also found correlations between population density and fewer barriers when seeking treatment with
145 respect to distance (r=0·61, p=0·02) and traveling alone (r=0·48, p=0·08); traveling alone is correlated
146 with distance (r=0·51, p<0·0001).

147 Coastal regions, namely, Dakar and Ziguinchor, have the highest female literacy rates (68% and 70%,
148 respectively). The percentage of women who received skilled prenatal care from a doctor, nurse, or
149 midwife was lowest in Tambacounda and Kedougou (88% and 85%, respectively), compared to over 95%
150 in other regions (Figure 1).

151 Poverty level did not statistically explain regional differences in DTP3 coverage (bivariate regression
152 coefficient β=-0·17, p=0·157), potentially due to heterogeneity within regions. We also found no
153 statistical associations between DTP3 coverage and the number of doctors, nurses, and midwives per
154 100,000 population using bivariate or multivariate regression analysis (Figure 3).

155 **Access to Health Services**

156 Women in regions with low DTP3 coverage reported more barriers to accessing care with respect to (i)
157 traveling alone to seek care and (ii) distance from healthcare facilities. In the more remote, inland regions
158 of Senegal, as many as 42% of female respondents in 2019 identified traveling alone to seek care as a
159 significant barrier to accessing care. Distance to healthcare facilities was also a substantial barrier to
160 accessing care in these regions of Senegal (identified as a barrier by 50% of female respondents in
161 Tambacounda, the largest region by geographical area). Sociodemographic characteristics of this survey
162 group are reported in the supplemental materials.

For peer review only - http://bmjopen.bmj.com/site/about/guidelines.xhtml

Coverage over time by geographic location

DTP3 coverage from 2005 to 2019 by region in Senegal is reported in Figure 4. We observed that the DTP3 coverage was consistently higher for the west and southwestern regions located near Dakar and along the coast, and the variance of DTP3 coverage over time was consistently lower (t-test, p=0.076) compared to that of the eastern and northern regions. The region with the lowest DTP3 coverage in 2019 was Kolda (73%), following a sharp drop in DTP3 coverage after 2017 compared to previous years. The rural and landlocked regions in the north and east demonstrated more variability in coverage over time. However, among these regions, Tambacounda and Kedougou saw large improvements, from 73% to 86% and 60% to 83%, respectively between 2017 and 2019; notably, Matam saw the largest increase (from 73% to 100%) in coverage among all regions between 2010 and 2019.

Discussion

We conducted a study to assess the demographic associations of early childhood vaccination within Senegal. Observing heterogeneities in DTP3 coverage across Senegal and comparing those differences with demographic and geographic factors may aid decision-makers in addressing disparities in vaccination access and uptake. Our analysis of household level data revealed associations between DTP3 coverage and 1) female literacy, 2) access to skilled prenatal care, and 3) challenges accessing care while traveling alone.

Early childhood vaccination in Senegal steadily increased for all recommended vaccines from 2002 to 2007, with coverage remaining mostly constant through 2019 [9, 24-29]. Based on an analysis of qualitative data including interviews with Senegalese administrators at various levels and focus group discussions with caregivers and community health workers, Senegal's immunization program has been successful due to "*strong governance, collaboration, evidence-based decision-making, community ownership, and an overall commitment to health and prioritization of vaccine programming from all stakeholders and government officials*."[9] Understanding the existing disparities between regions in

For peer review only - http://bmjopen.bmj.com/site/about/guidelines.xhtml

Senegal, alongside related factors, may support improvements in health equity through utilization of Senegal's existing governance structures and strong community health worker program.

*Female Literacy & Maternal Education***.** We found that higher female literacy was significantly associated with higher DTP3 coverage. Female literacy and higher maternal education have been shown to be positively associated with higher vaccination coverage globally [16, 30-32]; it is atypical that Senegal, with low female literacy at the national level, has relatively high DTP3 coverage compared to other LMICs. While mothers with high literacy and education may be more likely to independently seek vaccinations for their children [33], the health system in Senegal has been able to reach the more vulnerable rural communities with lower female literacy through community health workers and community-based health huts; "health huts are the backbone of Senegal's community health system, and they deserve much credit for Senegal's impressive progress on disease prevention and treatment, including its far-reaching immunization program" [7]. Involvement of community health workers such as respected female elders known as "bajenu gox" have served as a link between the health care services (i.e., health centers, health posts, health huts) and the communities, facilitating and coordinating immunization outreach activities [8].

*Access to Healthcare Workers & Facilities***.** We found that skilled prenatal care was associated with higher DTP3 coverage in Senegal, which aligns with existing literature. Access to skilled healthcare workers has an important role in childhood vaccination. Limited access to skilled prenatal care has been linked to under-vaccination, or children not receiving all recommended vaccines [13, 34], and a prior study highlighted an association between the number of skilled healthcare workers (nurses and midwives) and DTP3 coverage using a cross-country analysis of developing countries around the world for the years 1990 to 2004 [32]. Self-reported barriers limiting access to care for women when seeking treatment, namely traveling alone to healthcare facilities and travel distance, were associated with DTP3 vaccination. In regions with higher population density, women report fewer concerns regarding traveling

For peer review only - http://bmjopen.bmj.com/site/about/guidelines.xhtml

alone to access care when sick. Rural communities report more challenges than urban communities with respect to accessing immunization services. Unfortunately, retaining skilled health workers is more difficult in rural regions of Senegal [35]. Senegal has addressed these challenges through training community health workers and health post staff, improving infrastructure for outreach services, increasing capacity for health post expansion, and improving surveillance and data management at local levels [9]. In 2011, a 5-year $40 million grant from USAID helped Senegal construct, maintain, and link more health huts to the national health system [36].

Our results illustrate the role of contextual factors in relation to childhood vaccination. Although national childhood vaccination coverage in Senegal is relatively high compared to other LMICs, there are still gaps in coverage and disparities between regions which, similar to other studies, illustrate inequities in deployment of vaccination services [10, 11]. National health programs rely on the consideration of contextual factors and heterogeneity between sub-national areas, such as those identified in this study, to inform programmatic decision-making and improve health equity. Strategies for improving immunization rates need to be tailored considering subnational differences; as stated in The Immunization Agenda 2030, "reaching all people will require higher national vaccination coverage, but also less subnational inequity" [37]. Subnational childhood vaccination has been negatively correlated with institutional mistrust [38]. Strong governance structures are required to prevent and address institutional mistrust while prioritizing equity of healthcare access at the national level [9, 10, 39]. Sub-national DTP3 coverage estimates have been compared with national health spending, GDP per capita, and other governance indicators in sub-Saharan Africa to investigate the drivers of equitable immunization services [40]. To reduce differences in vaccination coverage between regions, and address institutional mistrust, national health policies and programs should consider heterogeneity between and within regions. Senegal has implemented top-down and bottom-up mechanisms for decision-making, planning, and resource allocation to address subnational heterogeneity and allow for strategies and initiatives tailored for local populations [9].

For peer review only - http://bmjopen.bmj.com/site/about/guidelines.xhtml

Conclusions

Senegal has improved vaccination coverage across its 14 regions despite high poverty and low female literacy as compared to other LICs and LMICs [17]. While coverage varies between regions, high coverage has expanded in recent years beyond coastal and urban regions into the inland and rural areas. National and regional efforts - especially increasing the number of health posts and huts to reach rural communities and expanding the community health worker programs - have likely played an important role [7]. Historically, the inland and rural regions have had variability in coverage over time; future years will tell if the recent increases in coverage will be sustained.

Limitations

This work focused on analyzing respondent groups at the regional level or higher, because DHS surveys were not sampled to be representative at the smaller district level. If district level data were available, we conjecture that negative associations between DTP3 coverage and other factors, such as poverty, could be identified, given heterogeneities at the subregional level.

List of abbreviations

DAH: Development assistance for health

DHS: Demographic and Health Surveys

DTP3: Third dose of the diphtheria-tetanus-pertussis vaccine

Gavi: Gavi, The Vaccine Alliance

GVAP: Global Vaccine Action Plan

LIC: Low income country

LMIC: Lower-middle income country

MSAS: Ministère de la Santé et de l’Action sociale

For peer review only - http://bmjopen.bmj.com/site/about/guidelines.xhtml

WHO: World Health Organization

Declarations

Ethics approval and consent to participate

Not applicable

Consent for publication

Not applicable

Availability of data and materials

The data that support the findings of this study are available at https://www.dhsprogram.com/data/available-datasets.cfm.

Competing interests

The authors declare that there are no competing interests.

Funding

Funding for this study was provided by the Bill & Melinda Gates Foundation, OPP1195041.

Authors' contributions

HS, FC, PK, and DN contributed to study structure. HS analyzed DHS and national data and HS, FC, PK, and DN synthesized key themes. ZS, MF, and MS provided essential context relating to qualitative factors within Senegal. All authors contributed to drafting the article and approve of the final manuscript.

Acknowledgements

This research was supported in part by the following Georgia Tech benefactors: William W. George, Andrea Laliberte, Joseph C. Mello, Richard "Rick" E. and Charlene Zalesky.

For peer review only - http://bmjopen.bmj.com/site/about/guidelines.xhtml

For peer review only

Figure legends

Figure 1 DTP3 coverage by region of Senegal and regional proportions of select metrics [41]. "Skilled Prenatal Care" refers to the proportion of women ages 15-49 who had a live birth within 5 years before the survey who had prenatal care provided by a skilled health care worker (2017). Skilled care includes doctor, nurse, and midwife. Unskilled care includes matron/traditional midwife, "other", or none. "Ease of Access to Care – Traveling Alone", refers to the proportion of female survey respondents by region who did not consider traveling alone to be a significant problem with accessing care when sick (2017).

Figure 2 Regression analysis for household-level factors associated with DTP3 vaccination by DHS 2019 survey response per living child aged 12-23 months (N=1183). The survey respondents per region represent equal proportions (0.10% of region population). The bars correspond to 95% confidence intervals. Bars which do not intersect 0 correspond to statistical associations with DTP3 vaccination (< 0 implies negative association, > 0 implies positive association).

Figure 3 Regression analysis for region-level factor associations with DTP3 vaccination. Poverty incidence is estimated using the cost of basic needs approach, which considers the proportion of the population unable to meet their basic needs and combines a country-specific food poverty line and non-food poverty line [42]. The bars correspond to 95% confidence intervals.

Figure 4 DTP3 coverage in Senegal by year and region (DHS 2005-2019). The regions of Kaffrine, Sedhiou, and Kedougou were split from existing regions in 2008 [43].

REFERENCES

1. Rémy V, Zöllner Y, Heckmann U. Vaccination: the cornerstone of an efficient healthcare system. J Mark Access Health Policy. 2015;3:10.3402/jmahp.v3.27041.
2. Ozawa S, Clark S, Portnoy A, Grewal S, Brenzel L, Walker DG. Return On Investment From Childhood Immunization In Low- And Middle-Income Countries, 2011-20. Health Aff (Millwood). 2016;35(2):199-207.

For peer review only

3. World Health Organization. Immunization coverage 2020 2021 [Available from: https://www.who.int/news-room/fact-sheets/detail/immunization-coverage.
4. World Health Organization. Global vaccine action plan 2011-2020 2013 [Available from: https://www.who.int/publications/i/item/global-vaccine-action-plan-2011-2020.
5. World Health Organization. 2018 assessment report of the Global Vaccine Action Plan: strategic advisory group of experts on immunization 2018 [Available from: https://apps.who.int/iris/handle/10665/276967.
6. World Health Organization. WHO vaccine-preventable diseases: monitoring system. 2020 global summary. WHO UNICEF estimates time series for Senegal (SEN) 2020 [Available from: https://apps.who.int/immunization_monitoring/globalsummary/estimates?c=SEN.
7. Exemplars in Global Health. What did Senegal do? 2021 [Available from: https://www.exemplars.health/topics/under-five-mortality/senegal/what-did-senegal-do.
8. Devlin K, Pandit-Rajani T, Egan KF. Senegal's Community-based Health System Model: Structure, Strategies, and Learning. Arlington, VA: Advancing Partners & Communities; 2019.
9. Sakas Z, Hester KA, Rodriguez K, Diatta SA, Ellis AS, Gueye DM, et al. Critical success factors for high routine immunization performance: A case study of Senegal. medRxiv. 2022:2022.01.25.22269847.
10. Faye CM, Wehrmeister FC, Melesse DY, Mutua MKK, Maïga A, Taylor CM, et al. Large and persistent subnational inequalities in reproductive, maternal, newborn and child health intervention coverage in sub-Saharan Africa. BMJ Global Health. 2020;5(1):e002232.
11. Mosser JF, Gagne-Maynard W, Rao PC, Osgood-Zimmerman A, Fullman N, Graetz N, et al. Mapping diphtheria-pertussis-tetanus vaccine coverage in Africa, 2000–2016: a spatial and temporal modelling study. The Lancet. 2019;393(10183):1843-55.
12. Utazi CE, Thorley J, Alegana VA, Ferrari MJ, Takahashi S, Metcalf CJE, et al. High resolution age-structured mapping of childhood vaccination coverage in low and middle income countries. Vaccine. 2018;36(12):1583-91.
13. Eze P, Agu UJ, Aniebo CL, Agu SA, Lawani LO, Acharya Y. Factors associated with incomplete immunisation in children aged 12–23 months at subnational level, Nigeria: a cross-sectional study. BMJ Open. 2021;11(6):e047445.
14. Sarker AR, Akram R, Ali N, Chowdhury ZI, Sultana M. Coverage and Determinants of Full Immunization: Vaccination Coverage among Senegalese Children. Medicina. 2019;55(8).
15. Ameyaw EK, Kareem YO, Ahinkorah BO, Seidu A-A, Yaya S. Decomposing the rural–urban gap in factors associated with childhood immunisation in sub-Saharan Africa: evidence from surveys in 23 countries. BMJ Global Health. 2021;6(1):e003773.
16. Mbengue MAS, Sarr M, Faye A, Badiane O, Camara FBN, Mboup S, et al. Determinants of complete immunization among senegalese children aged 12-23 months: evidence from the demographic and health survey. BMC Public Health. 2017;17(1):630.
17. Bednarczyk RA, Hester KA, Dixit SM, Ellis AS, Escoffery C, Kilembe W, et al. Exemplars in vaccine delivery protocol: a case-study-based identification and evaluation of critical factors in achieving high and sustained childhood immunisation coverage in selected low-income and lower-middle-income countries. BMJ Open. 2022;12(4):e058321.
18. Hester KA, Sakas Z, Ellis AS, Bose AS, Darwar R, Gautam J, et al. Critical success factors for high routine immunization performance: A case study of Nepal. Vaccine: X. 2022;12:100214.
19. Micek K, Hester KA, Chanda C, Darwar R, Dounebaine B, Ellis AS, et al. Critical success factors for routine immunization performance: A case study of Zambia 2000 to 2018. Vaccine: X. 2022;11:100166.
20. The DHS Program. Senegal Continuous Demographic Health Survey 2005-2019 2019 [Available from: https://www.dhsprogram.com/data/available-datasets.cfm.
21. Knoema. Senegal 2021 [Available from: https://knoema.com/atlas/Senegal.

For peer review only - http://bmjopen.bmj.com/site/about/guidelines.xhtml

22. Ministère de la Santé et de l'Action sociale. Annuaire des statistiques sanitaires et sociales 2018 2020 [Available from: https://sante.sec.gouv.sn/sites/default/files/Annuaire%20Satatistiques%20sanitaires%20et%20sociales%202018.pdf.
23. Senegal Data Portal. Population & Financial Statistics of Senegal, 2011 2012 [Available from: https://senegal.opendataforafrica.org/SEPFS2016/population-financial-statistics-of-senegal-2011.
24. WHO and UNICEF. Senegal: WHO and UNICEF estimates of immunization coverage: 2020 revision 2021 [Available from: https://cdn.who.int/media/docs/default-source/country-profiles/immunization/immunization_sen_2021.pdf.
25. Unicef. Immunization data July 2021 2021 [Available from: https://data.unicef.org/resources/dataset/immunization/.
26. Russo G, Lihui X, McIsaac M, Matsika-Claquin MD, Dhillon I, McPake B, et al. Health workers' strikes in low-income countries: the available evidence. Bulletin of the World Health Organization. 2019.
27. HRH2030 Program. Three Questions with Dr. Matar Camara: A public health specialist with a vision 2018 [Available from: https://hrh2030program.org/3-questions-with-matar-camara-2/.
28. Despite Senegal's Nine-Month Health Worker Strike, Nioro Sees LARC Usage Trend Improve: TCI: The Challenge Initiative; 2020 [Available from: https://tciurbanhealth.org/despite-senegals-nine-month-health-worker-strike-nioro-sees-positive-impact-from-larcs/.
29. WHO and UNICEF. Senegal: WHO and UNICEF estimates of immunization coverage: 2019 revision 2020 [Available from: https://www.who.int/immunization/monitoring_surveillance/data/sen.pdf.
30. Streatfield K, Singarimbun M, Diamond I. Maternal Education and Child Immunization. Demography. 1990;27(3):447-55.
31. Vikram K, Vanneman R, Desai S. Linkages between maternal education and childhood immunization in India. Soc Sci Med. 2012;75(2):331-9.
32. Anand S, Bärnighausen T. Health workers and vaccination coverage in developing countries: an econometric analysis. The Lancet. 2007;369(9569):1277-85.
33. Balogun SA, Yusuff HA, Yusuf KQ, Al-Shenqiti AM, Balogun MT, Tettey P. Maternal education and child immunization: the mediating roles of maternal literacy and socioeconomic status. (1937-8688 (Electronic)).
34. Rainey JJ, Watkins M, Ryman TK, Sandhu P, Bo A, Banerjee K. Reasons related to non-vaccination and under-vaccination of children in low and middle income countries: Findings from a systematic review of the published literature, 1999–2009. Vaccine. 2011;29(46):8215-21.
35. Zurn P, Codjia L Fau - Sall FL, Sall Fl Fau - Braichet J-M, Braichet JM. How to recruit and retain health workers in underserved areas: the Senegalese experience. Bull World Health Organ.88(5):386-9.
36. ChildFund. Senegal's Commitment to Community Health Yields Benefits 2021 [Available from: https://www.childfund.org/senegals-commitment-to-community-health/.
37. World Health Organization. Immunization Agenda 2030: A Global Strategy to Leave No One Behind. World Health Organization; 2020.
38. Stoop N, Hirvonen K, Maystadt J-F. Institutional mistrust and child vaccination coverage in Africa. BMJ Global Health. 2021;6(4):e004595.
39. Shen AK, Fields R, McQuestion M. The future of routine immunization in the developing world: challenges and opportunities. Glob Health Sci Pract. 2014;2(4):381-94.
40. Ikilezi G, Augusto OJ, Sbarra A, Sherr K, Dieleman JL, Lim SS. Determinants of geographical inequalities for DTP3 vaccine coverage in sub-Saharan Africa. Vaccine. 2020;38(18):3447-54.
41. Agence Nationale de la Statistique et de la Démographie (ANSD). Sénégal : Enquête Démographique et de Santé Continue (EDS-Continue) 2019 2020 [Available from: https://dhsprogram.com/pubs/pdf/FR368/FR368.T.pdf.

For peer review only - http://bmjopen.bmj.com/site/about/guidelines.xhtml

42. Agence Nationale de la Statistique et de la Démographie (ANSD). Deuxieme Enquete De Suivi De La Pauvrete Au Senegal (ESPS-11 2011) 2013 [Available from: https://www.ansd.sn/ressources/publications/Rapport_ESPS-2011.pdf.

43. Monkam NF. Property Taxation in Senegal: Legislation and Practice. Journal of Property Tax Assessment & Administration. 2011;8(3):41-60.

For peer review only

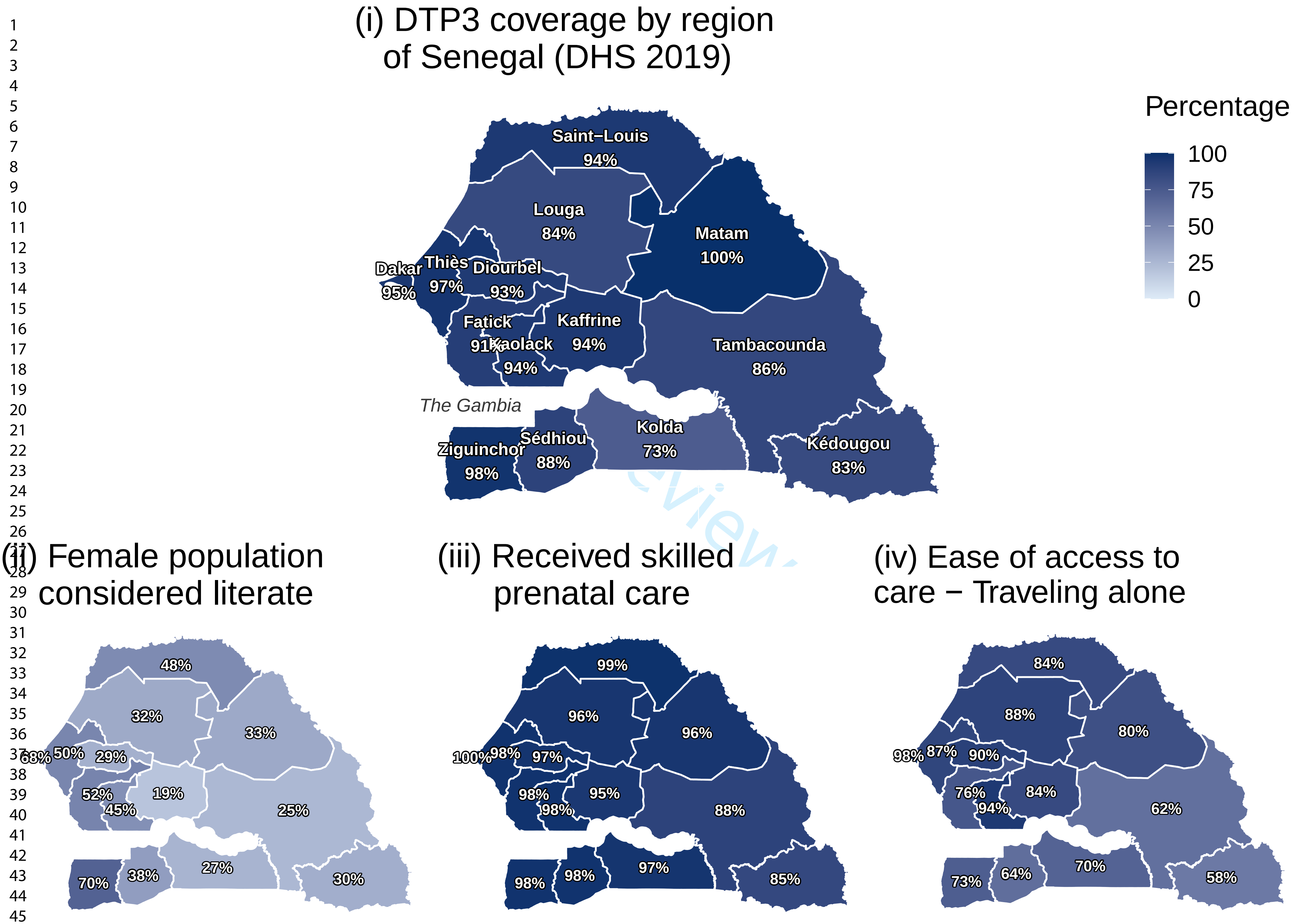
(i) DTP3 coverage by region of Senegal (DHS 2019)
Percentage
100
75
50
25
0
Saint−Louis 94%
Louga 84%
Matam 100%
Dakar 95%
Thiès 97%
Diourbel 93%
Fatick 91%
Kaolack 94%
Kaffrine 94%
Tambacounda 86%
The Gambia
Ziguinchor 98%
Sédhiou 88%
Kolda 73%
Kédougou 83%
(ii) Female population considered literate
48%
32%
33%
68%
50%
29%
52%
45%
19%
25%
70%
38%
27%
30%
(iii) Received skilled prenatal care
99%
96%
96%
100%
98%
97%
98%
98%
95%
88%
98%
98%
97%
85%
(iv) Ease of access to care − Traveling alone
84%
88%
80%
98%
87%
90%
76%
94%
84%
62%
73%
64%
70%
58%

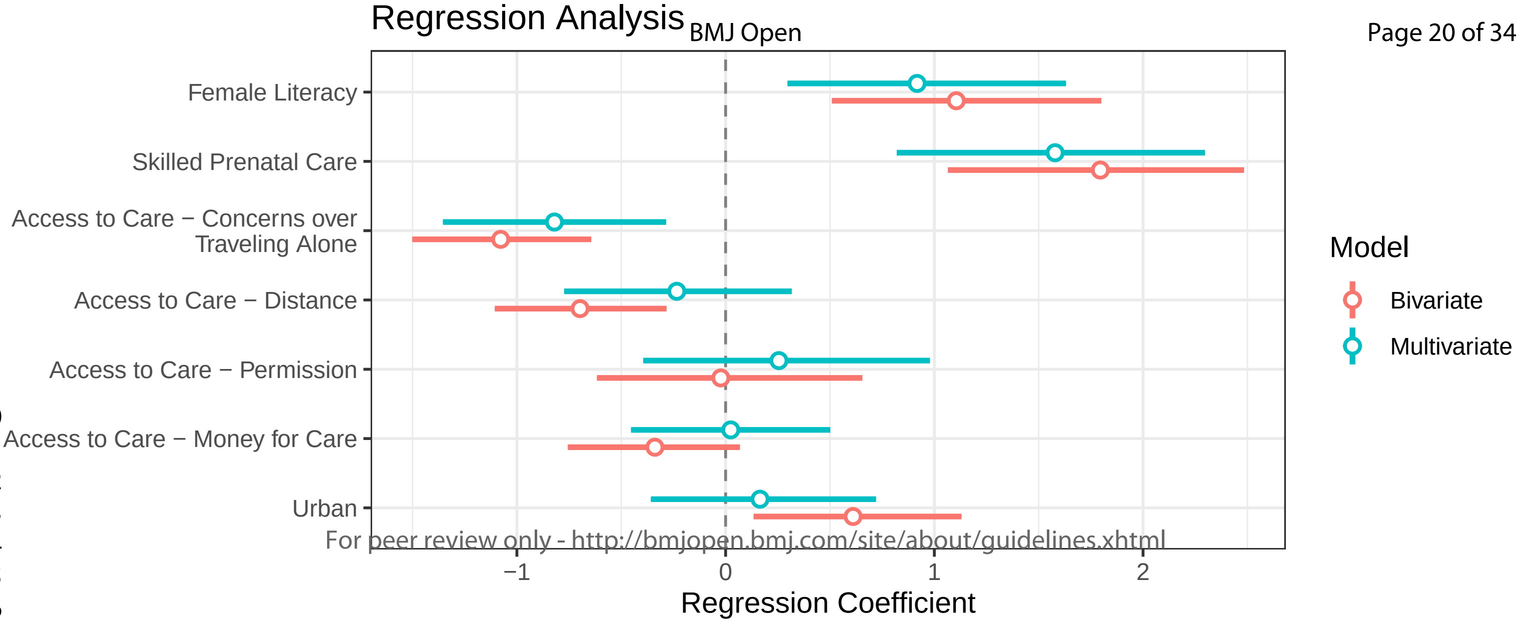

For peer review only - http://bmjopen.bmj.com/site/about/guidelines.xhtml

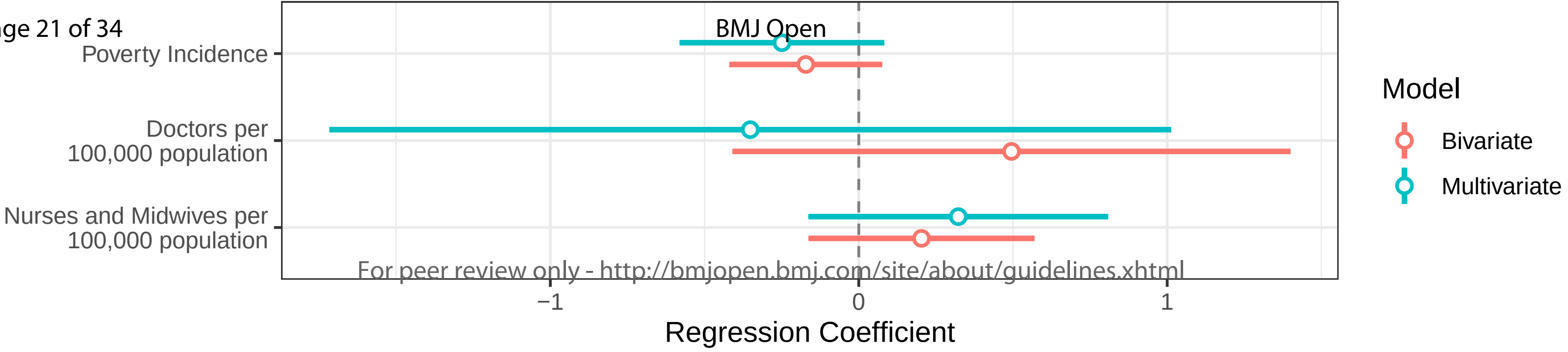

For peer review only - http://bmjopen.bmj.com/site/about/guidelines.xhtml

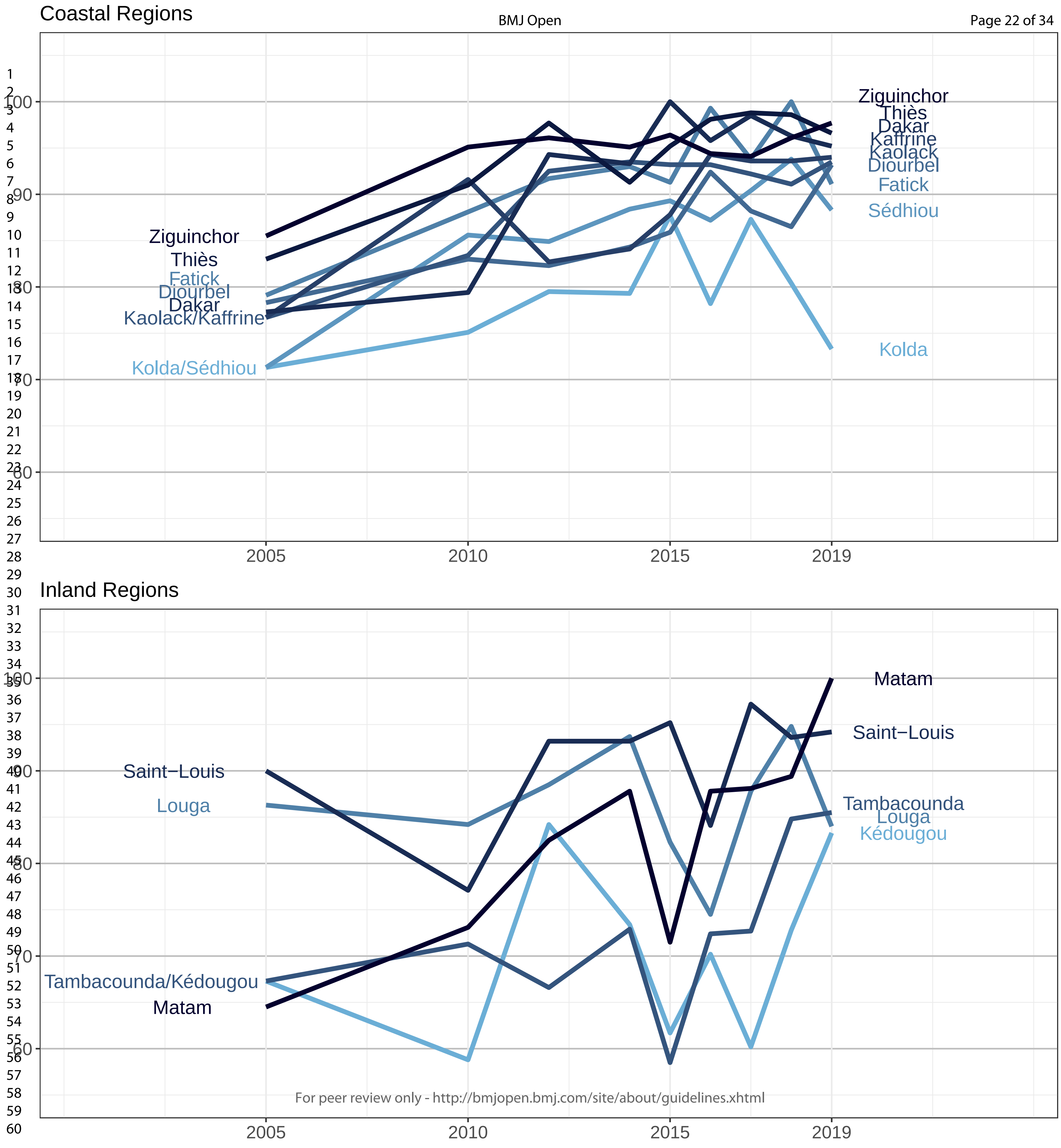
Coastal Regions
100
90
80
70
60
2005
2010
2015
2019
Ziguinchor
Thiès
Fatick
Diourbel
Dakar
Kaolack/Kaffrine
Kolda/Sédhiou
Ziguinchor
Thiès
Dakar
Kaffrine
Kaolack
Diourbel
Fatick
Sédhiou
Kolda
Inland Regions
100
90
80
70
60
2005
2010
2015
2019
Saint−Louis
Louga
Tambacounda/Kédougou
Matam
Matam
Saint−Louis
Tambacounda
Louga
Kédougou

Section 1: Comparing Senegal and other lower income and lower-middle income countries

Senegal is considered an Exemplar country as part of the Exemplars in Global Health program, having made "extraordinary progress in important health outcomes" [1]. Based on Exemplar selection criteria [1] and a cutoff of US$1025 per capita between lower income (LIC) and lower-middle income (LMIC) in 2018, countries are separated into four groups: exemplar LIC, non-exemplar LIC, exemplar LMIC, and non-exemplar LMIC [2, 3]. Senegal has been identified as an exemplar, and in 2018, Senegal had a GNI per capita of $1410, indicating LMIC status. To determine Senegal's position relative to other LICs and LMICs, we compared Senegal with other LIC and LMIC and exemplar and non-exemplar countries by observing trends over time for Development Assistance for Health (DAH) using data from the Institute for Health Metrics and Evaluation (IHME, 2000-2017) [4] and WHO-UNICEF [5], and female literacy (2000-2017) using data from World Bank [6]. Values are presented for each country and also averaged across countries by group.

Figure 1 reports the DTP3 coverage for Senegal and the other exemplar and non-exemplar countries over time; note that group trends are yearly averages. Senegal is performing near the top of the group with respect to coverage with 5 years persistently above 90% coverage.

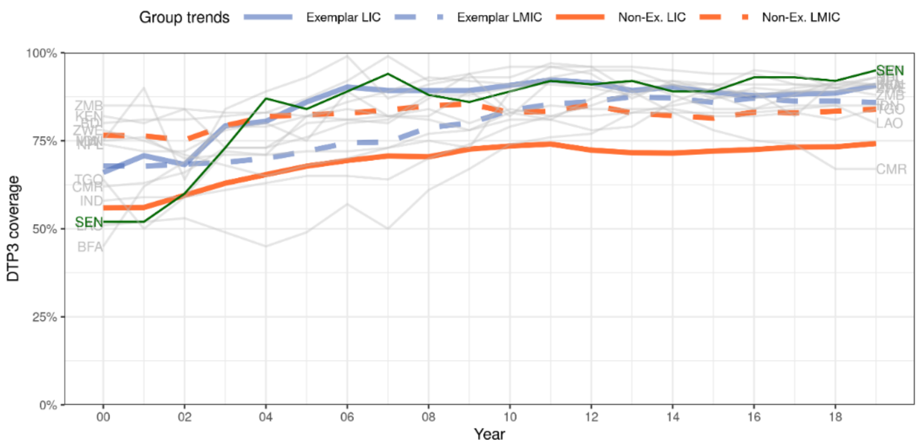

Figure 1 DTP3 coverage for Senegal and other LICs and LMICs and exemplar/non-exemplar groups, 2000-2019.

Development assistance for health (DAH) per birth on newborn and child health vaccines for Senegal is reported in *Figure 2*. External funding for newborn and childhood vaccines increased substantially in 2013. This increase

For peer review only - http://bmjopen.bmj.com/site/about/guidelines.xhtml

coincides with Gavi support for PCV13 (October 2013) and MCV (introduction of measles-rubella and measles dose 2, 2014) [7].

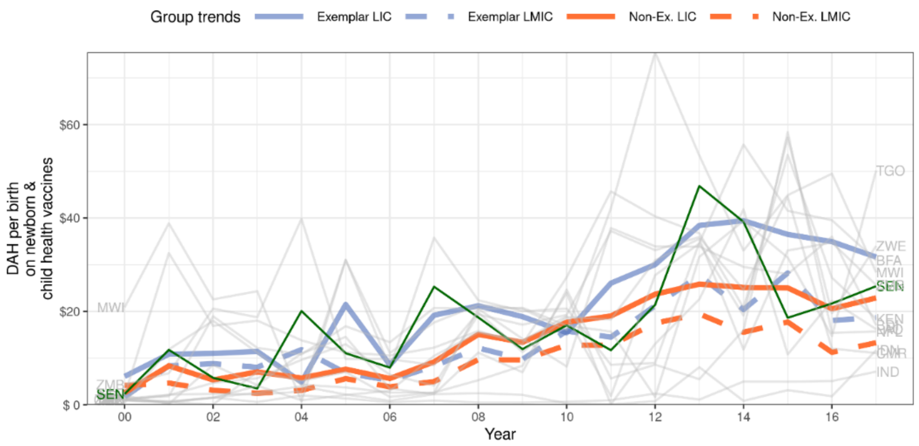

Figure 2 DAH per birth on newborn and child health vaccines for Senegal and LICs/LMICs, 2000-2017

Senegal's literacy rate among women is low compared to the other LICs and LMICs analyzed, with an upward trend but increasing slower than other exemplar and non-exemplar countries (*Figure 3*) [6].

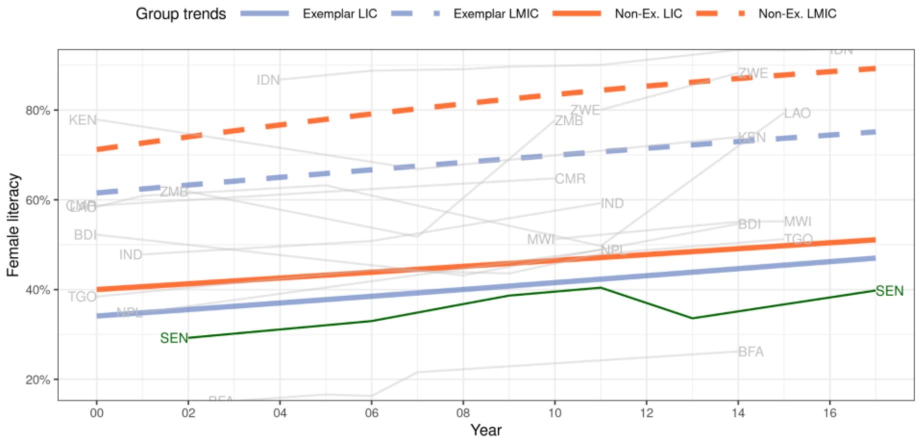

Figure 3 Female Literacy in Senegal Compared to Exemplar and Non-Exemplar LICs and LMICs

For peer review only - http://bmjopen.bmj.com/site/about/guidelines.xhtml

Section 2: Additional tables

**Variable**	**β Coefficients and 95% CI**	**p-value**
Urban	0·16 (-0·36, 0·72)	0·547
**Female Literacy**	**0·92 (0·30, 1·63)**	**0·0065**
**Skilled Prenatal Care from a doctor, nurse, or midwife**	**1·58 (0·82, 2·30)**	**< 0·0001**
Problems experienced among respondents who sought care when sick: Women ages 15-49 were asked if the following were significant barriers when seeking treatment when they were sick.		
Access to Care - Distance	-0·23 (-0·77, 0·32)	0·400
Access to Care - Permission	0·26 (-0·39, 0·98)	0·463
**Access to Care - Concerns over Traveling Alone**	**-0·82 (-1·35, -0·28)**	**0·0026**
Access to Care - Money for Care	0·02 (-0·45, 0·50)	0·919

Table 1 Household-level factors associated with DTP3 vaccination by survey response per living child age 12-23 months (N=1183): multivariate regression analysis. DHS 2019. The survey respondents per region represent equal proportions (0.10% of region population).

For peer review only

Variable	Coefficients and 95% CI	p-value
Poverty Incidence	-0·249 (-0·58, 0·08)	0·126
Doctors per 100,000 population	-0·352 (-1·72, 1·01)	0·579
Nurses and Midwives per 100,000 population	0·323 (-0·16, 0·81)	0·170

Table 2 Region-level factor associations with DTP3 coverage at the regional level (N=14): multivariate linear regression analysis. DHS 2019. Poverty incidence is estimated using the cost of basic needs approach, which considers the proportion of the population unable to meet their basic needs and combines a country-specific food poverty line and non-food poverty line. For more details with respect to this calculation specific to Senegal, see [8].

For peer review only - http://bmjopen.bmj.com/site/about/guidelines.xhtml

Sociodemographic Characteristics of Survey Respondents (n=8,649)	
**Religion**	
Muslim	97%
Christian	3%
**Ethnicity**	
Wolof	40%
Poular	28%
Serer	15%
Mandingue/Soce	5%
Diola	4%
Soninke	3%
Other/non-Senegalese	5%
**Education**	
Primary	21%
Secondary or more	32%
**Marital Status**	
Single	30%
Married	65%
Divorced/separated	4%
Widow	1%
**Age**	
15-49	22%
20-24	19%
25-29	16%
30-34	15%
35-39	13%
40-44	8%
45-49	7%

Table 3 Sociodemographic characteristics of female survey respondents, 2019.[9]

For peer review only

For peer review only - http://bmjopen.bmj.com/site/about/guidelines.xhtml

REFERENCES

1. Exemplars in Global Health. Leading by example: a resource for global health decision makers 2021 [Available from: https://www.exemplars.health/learn-more/about.
2. Bednarczyk RA, Hester KA, Dixit SM, Ellis AS, Escoffery C, Kilembe W, et al. Protocol: Identification and evaluation of critical factors in achieving high and sustained childhood immunization coverage in selected low- and lower-middle income countries. medRxiv. 2021:2021.12.01.21267018.
3. Castillo-Zunino F, Keskinocak P, Nazzal D, Freeman MC. Health spending and vaccination coverage in low-income countries. BMJ Global Health. 2021;6(5):e004823.
4. IHME. Global health spending 1995-2016 2019 [Available from: http://ghdx.healthdata.org/record/ihme-data/global-health-spending-1995-2016
5. WHO and UNICEF. WHO-UNICEF joint reporting form: immunization financing indicators, 2019 [Available from: https://www.who.int/immunization/programmes_systems/financing/data_indicators/en/.
6. The World Bank. Literacy rate, adult female 2020 [Available from: https://data.worldbank.org/indicator/SE.ADT.LITR.FE.ZS
7. Gavi Vaccine Alliance. Senegal 2020 [Available from: https://www.gavi.org/programmes-impact/country-hub/africa/senegal.
8. Agence Nationale de la Statistique et de la Démographie (ANSD). Deuxieme Enquete De Suivi De La Pauvrete Au Senegal (ESPS-11 2011) 2013 [Available from: https://www.ansd.sn/ressources/publications/Rapport_ESPS-2011.pdf.
9. Agence Nationale de la Statistique et de la Démographie (ANSD). Sénégal : Enquête Démographique et de Santé Continue (EDS-Continue) 2019 2020 [Available from: https://dhsprogram.com/pubs/pdf/FR368/FR368.T.pdf.

For peer review only - http://bmjopen.bmj.com/site/about/guidelines.xhtml

The RECORD statement – checklist of items, extended from the STROBE statement, that should be reported in observational studies using routinely collected health data.

	Item No.	STROBE items	Location in manuscript where items are reported	RECORD items	Location in manuscript where items are reported
**Title and abstract**					
	1	(a) Indicate the study's design with a commonly used term in the title or the abstract (b) Provide in the abstract an informative and balanced summary of what was done and what was found	(a) Abstract: Methods<br>(b) Abstract: Methods and Results<br>(page 2)	RECORD 1.1: The type of data used should be specified in the title or abstract. When possible, the name of the databases used should be included.<br>RECORD 1.2: If applicable, the geographic region and timeframe within which the study took place should be reported in the title or abstract.<br>RECORD 1.3: If linkage between databases was conducted for the study, this should be clearly stated in the title or abstract.	Abstract: Methods (page 2)
**Introduction**					
Background rationale	2	Explain the scientific background and rationale for the investigation being reported	Page 5		
Objectives	3	State specific objectives, including any prespecified hypotheses	Page 5		
**Methods**					
Study Design	4	Present key elements of study design early in the paper	Page 5-6		
Setting	5	Describe the setting, locations, and relevant dates, including periods of recruitment, exposure, follow-up, and data collection	Page 6		

For peer review only

Participants	6	*(a) Cohort study* - Give the eligibility criteria, and the sources and methods of selection of participants. Describe methods of follow-up<br>*Case-control study* - Give the eligibility criteria, and the sources and methods of case ascertainment and control selection. Give the rationale for the choice of cases and controls<br>*Cross-sectional study* - Give the eligibility criteria, and the sources and methods of selection of participants<br>*(b) Cohort study* - For matched studies, give matching criteria and number of exposed and unexposed<br>*Case-control study* - For matched studies, give matching criteria and the number of controls per case	Data analysis – page 7. Retrospective analysis – living children aged 12-23 months from DHS reports for each year.	RECORD 6.1: The methods of study population selection (such as codes or algorithms used to identify subjects) should be listed in detail. If this is not possible, an explanation should be provided.<br>RECORD 6.2: Any validation studies of the codes or algorithms used to select the population should be referenced. If validation was conducted for this study and not published elsewhere, detailed methods and results should be provided.<br>RECORD 6.3: If the study involved linkage of databases, consider use of a flow diagram or other graphical display to demonstrate the data linkage process, including the number of individuals with linked data at each stage.	Data analysis – page 7. Retrospective analysis – living children aged 12-23 months from DHS reports for each year.
Variables	7	Clearly define all outcomes, exposures, predictors, potential confounders, and effect modifiers. Give diagnostic criteria, if applicable.	Data sources and data analysis, pages 6-7	RECORD 7.1: A complete list of codes and algorithms used to classify exposures, outcomes, confounders, and effect modifiers should be provided. If these cannot be reported, an explanation should be provided.	Data sources and data analysis, pages 6-7
Data sources/ measurement	8	For each variable of interest, give sources of data and details of methods of assessment (measurement).	Data sources and data analysis, pages 6-7		

For peer review only

		Describe comparability of assessment methods if there is more than one group			
Bias	9	Describe any efforts to address potential sources of bias	Data analysis, page 7		
Study size	10	Explain how the study size was arrived at	All available DHS data for Senegal; data sources page 6		
Quantitative variables	11	Explain how quantitative variables were handled in the analyses. If applicable, describe which groupings were chosen, and why	Data sources and data analysis, page 6-7		
Statistical methods	12	(a) Describe all statistical methods, including those used to control for confounding<br>(b) Describe any methods used to examine subgroups and interactions<br>(c) Explain how missing data were addressed<br>(d) *Cohort study* - If applicable, explain how loss to follow-up was addressed<br>*Case-control study* - If applicable, explain how matching of cases and controls was addressed<br>*Cross-sectional study* - If applicable, describe analytical methods taking account of sampling strategy<br>(e) Describe any sensitivity analyses	Data analysis, page 7		

For peer review only

Data access and cleaning methods		…		RECORD 12.1: Authors should describe the extent to which the investigators had access to the database population used to create the study population.<br>RECORD 12.2: Authors should provide information on the data cleaning methods used in the study.	Retrospective analysis of DHS data
Linkage		…		RECORD 12.3: State whether the study included person-level, institutional-level, or other data linkage across two or more databases. The methods of linkage and methods of linkage quality evaluation should be provided.	Retrospective analysis of DHS data
**Results**					
Participants	13	(a) Report the numbers of individuals at each stage of the study (*e.g.*, numbers potentially eligible, examined for eligibility, confirmed eligible, included in the study, completing follow-up, and analysed)<br>(b) Give reasons for non-participation at each stage.<br>(c) Consider use of a flow diagram	Figure 2 caption reports included 2019 survey respondents. 14 regions considered in region-level analysis and stated throughout.	RECORD 13.1: Describe in detail the selection of the persons included in the study (*i.e.,* study population selection) including filtering based on data quality, data availability and linkage. The selection of included persons can be described in the text and/or by means of the study flow diagram.	Data analysis – page 7. Retrospective analysis – living children aged 12-23 months from DHS reports for each year.
Descriptive data	14	(a) Give characteristics of study participants (*e.g.*, demographic, clinical, social) and information on exposures and potential confounders<br>(b) Indicate the number of participants with missing data for each variable of interest	Supplemental materials Table 3		

For peer review only

		(c) *Cohort study* - summarise follow-up time (*e.g.*, average and total amount)			
Outcome data	15	*Cohort study* - Report numbers of outcome events or summary measures over time<br>*Case-control study* - Report numbers in each exposure category, or summary measures of exposure<br>*Cross-sectional study* - Report numbers of outcome events or summary measures	Throughout results		
Main results	16	(a) Give unadjusted estimates and, if applicable, confounder-adjusted estimates and their precision (e.g., 95% confidence interval). Make clear which confounders were adjusted for and why they were included<br>(b) Report category boundaries when continuous variables were categorized<br>(c) If relevant, consider translating estimates of relative risk into absolute risk for a meaningful time period	In main body results and supplemental materials Tables 1 and 2.		
Other analyses	17	Report other analyses done—e.g., analyses of subgroups and interactions, and sensitivity analyses	In main body results.		
**Discussion**					
Key results	18	Summarise key results with reference to study objectives	Beginning of Discussion page 13		

For peer review only

Limitations	19	Discuss limitations of the study, taking into account sources of potential bias or imprecision. Discuss both direction and magnitude of any potential bias	End of page 14	RECORD 19.1: Discuss the implications of using data that were not created or collected to answer the specific research question(s). Include discussion of misclassification bias, unmeasured confounding, missing data, and changing eligibility over time, as they pertain to the study being reported.	End of page 14
Interpretation	20	Give a cautious overall interpretation of results considering objectives, limitations, multiplicity of analyses, results from similar studies, and other relevant evidence	Throughout discussion		
Generalisability	21	Discuss the generalisability (external validity) of the study results	Discussion page 15		
**Other Information**					
Funding	22	Give the source of funding and the role of the funders for the present study and, if applicable, for the original study on which the present article is based	Page 17		
Accessibility of protocol, raw data, and programming code		..		RECORD 22.1: Authors should provide information on how to access any supplemental information such as the study protocol, raw data, or programming code.	Page 17

*Reference: Benchimol EI, Smeeth L, Guttmann A, Harron K, Moher D, Petersen I, Sørensen HT, von Elm E, Langan SM, the RECORD Working Committee. The REporting of studies Conducted using Observational Routinely-collected health Data (RECORD) Statement. *PLoS Medicine* 2015; in press.

For peer review only

*Checklist is protected under Creative Commons Attribution (CC BY) license.

For peer review only

BMJ Open

Factors associated with vaccine coverage improvements in Senegal between 2005-2019: A quantitative retrospective analysis

Journal:	*BMJ Open*
Manuscript ID	bmjopen-2023-074388.R1
Article Type:	Original research
Date Submitted by the Author:	26-Jul-2023
Complete List of Authors:	Smalley, Hannah; Georgia Institute of Technology, H. Milton Stewart School of Industrial and Systems Engineering<br>Castillo-Zunino, Francisco; Amazon, Amazon Modeling and Optimization<br>Keskinocak, Pinar; Georgia Institute of Technology, ISYE<br>Nazzal, Dima; Georgia Institute of Technology, Industrial and Systems Engineering<br>Sakas, Zoë; Emory University, Gangarosa Department of Environmental Health, Rollins School of Public Health<br>Sarr, Moussa; Institut de Recherche en Santé de Surveillance Epidemiologique et de Formation<br>Freeman, Matthew; Emory Univ, Gangarosa Department of Environmental Health
**Primary Subject Heading**:	Public health
Secondary Subject Heading:	Health policy, Global health, Paediatrics
Keywords:	Health policy < HEALTH SERVICES ADMINISTRATION & MANAGEMENT, Public health < INFECTIOUS DISEASES, Paediatric infectious disease & immunisation < PAEDIATRICS

SCHOLARONE™
Manuscripts

BMJ

I, the Submitting Author has the right to grant and does grant on behalf of all authors of the Work (as defined in the below author licence), an exclusive licence and/or a non-exclusive licence for contributions from authors who are: i) UK Crown employees; ii) where BMJ has agreed a CC-BY licence shall apply, and/or iii) in accordance with the terms applicable for US Federal Government officers or employees acting as part of their official duties; on a worldwide, perpetual, irrevocable, royalty-free basis to BMJ Publishing Group Ltd ("BMJ") its licensees and where the relevant Journal is co-owned by BMJ to the co-owners of the Journal, to publish the Work in this journal and any other BMJ products and to exploit all rights, as set out in our licence.

The Submitting Author accepts and understands that any supply made under these terms is made by BMJ to the Submitting Author unless you are acting as an employee on behalf of your employer or a postgraduate student of an affiliated institution which is paying any applicable article publishing charge ("APC") for Open Access articles. Where the Submitting Author wishes to make the Work available on an Open Access basis (and intends to pay the relevant APC), the terms of reuse of such Open Access shall be governed by a Creative Commons licence – details of these licences and which Creative Commons licence will apply to this Work are set out in our licence referred to above.

Other than as permitted in any relevant BMJ Author's Self Archiving Policies, I confirm this Work has not been accepted for publication elsewhere, is not being considered for publication elsewhere and does not duplicate material already published. I confirm all authors consent to publication of this Work and authorise the granting of this licence.

For peer review only

Factors associated with vaccine coverage improvements in Senegal between 2005-2019: A quantitative retrospective analysis

Hannah K Smalley[1], Francisco Castillo-Zunino[2i], Pinar Keskinocak,[1,3], Dima Nazzal[1], Zoe Sakas[4], Moussa Sarr[5], Matthew C. Freeman[4]*

1 H. Milton Stewart School of Industrial and Systems Engineering, Georgia Institute of Technology, 755 Ferst Dr., NW, Atlanta, GA, USA.

2 Amazon Modeling and Optimization, Amazon, Bellevue, WA, USA.

3 Center for Health and Humanitarian Systems, Georgia Institute of Technology, 755 Ferst Dr., NW, Atlanta, GA, USA.

4 Gangarosa Department of Environmental Health, Rollins School of Public Health, Emory University, Atlanta, GA, USA

5 Institut de Recherche en Santé de Surveillance Epidemiologique et de Formation, Dakar, Senegal

* Corresponding author's email address: hannahsmalley@gatech.edu

[i] Work performed while the author was with Georgia Institute of Technology prior to joining Amazon.

For peer review only - http://bmjopen.bmj.com/site/about/guidelines.xhtml

ABSTRACT

Objective Senegal has demonstrated catalytic improvements in national coverage rates for early childhood vaccination, despite lower development assistance for childhood vaccines in Senegal compared to other low and lower-middle income countries. Understanding factors associated with historical changes in childhood vaccine coverage in Senegal, as well as heterogeneities across its 14 regions, can highlight effective practices that might be adapted to improve vaccine coverage elsewhere.

Design Childhood vaccination coverage rates, demographic information, and health system characteristics were identified from Senegal's Demographic and Health Surveys (DHS) and Senegal national reports for years 2005 to 2019. Multivariate logistic and linear regression analyses were performed to determine statistical associations of demographic and health system characteristics with respect to childhood vaccination coverage rates.

Setting The 14 administrative regions of Senegal were chosen for analysis.

Participants DHS women's survey respondents with living children aged 12-23 months for survey years 2005-2019.

Outcome Measures Immunization with the third dose of the diphtheria-tetanus-pertussis vaccine (DTP3), widely used as a proxy for estimating immunization coverage levels and the retention of children in the vaccine program.

Results Factors associated with childhood vaccination coverage include urban residence (β=0·61, p=0·0157), female literacy (β=1·11, p=0·0007),, skilled prenatal care (β=1·80, p<0·0001), and self-reported ease of access to care when sick, considering travel distance to a healthcare facility (β=-0·70, p=0·0009) and concerns over traveling alone (β=-1·08, p<0·0001). Higher coverage with less variability over time was reported in urban areas near the capital and the coast (p=0·076), with increased coverage in recent years in more rural and landlocked areas.

For peer review only - http://bmjopen.bmj.com/site/about/guidelines.xhtml

Conclusions Childhood vaccination was more likely among children whose mothers had higher literacy, received skilled prenatal care, and had perceived ease of access to care when sick. Overall, vaccination coverage is high in Senegal and disparities in coverage between regions have decreased significantly in recent years.

Strengths and limitations of the study

- This study explains associations between DTP3 coverage and demographic and health system characteristics at the subnational level within Senegal.
- This study evaluates changes in vaccination coverage over time by administrative region and geographic area.
- Analysis was not performed at the district level and thus heterogeneities at the subregional level were not evaluated.

Keywords: Childhood immunization coverage; Vaccines; Senegal; Inequality; Regression analysis

Background

Vaccination is one of the most cost-effective and influential public health interventions [1]. For each dollar spent on routine and supplementary vaccinations in low- and middle-income countries from 2011-2020, the average country-level return on investment is estimated to be 44 times the cost [2]. High vaccination coverage is necessary for controlling, eliminating, or eradicating vaccine-preventable diseases [3]. Gavi, The Vaccine Alliance (Gavi), was launched in 2000 and is one of the main sources of vaccine financing for eligible low- and middle-income countries. Some but not all Gavi-supported countries were able to reach the 2015 Global Vaccine Action Plan (GVAP) goal of 90% coverage for the third dose of the diphtheria-tetanus-pertussis vaccine (DTP3) by 2010 [4, 5].

For peer review only - http://bmjopen.bmj.com/site/about/guidelines.xhtml

In 2017, Africa reported a DTP3 coverage of 72%, the lowest percentage of all the World Health Organization (WHO) regions [4]. Within the African Region, several countries have outperformed their peers with regards to high and sustained vaccination coverage. DTP3 coverage in Senegal increased from 52% to 93% [6], outperforming other low income (LIC) and lower-middle income (LMIC) countries, despite lower development assistance for health (DAH) relative to many other countries in this group [7, 8], . The Government of Senegal has demonstrated a commitment to improving access to health care, including childhood vaccination, which likely contributed to the country's success [9-11]. However, like other countries in sub-Saharan Africa, Senegal's childhood vaccine coverage was not consistent between Senegal's 14 administrative regions

The purpose of this study was to identify factors associated with DTP3 coverage in Senegal at both the household level and regionally. DTP3 is often used as a proxy for estimating the retention of children in the vaccine program [3]. Subnational trends in early childhood vaccine coverage often illustrate inequities in vaccine service delivery [12, 13], and looking at national-level data alone may overestimate vaccine system performance [14]. Factors statistically associated with full childhood immunization coverage have been identified in previous studies [15-18]. We complement prior work by focusing on DTP3 coverage in Senegal, with an analysis of changes in coverage over time by administrative region from 2005-2019. We analyzed geographic and population factors, including female literacy and access to skilled prenatal care. We also considered factors aggregated at the regional level including poverty and the number of healthcare workers.

Methods

We conducted a study to assess the demographic associations of early childhood vaccination within Senegal, including assessing the impact of heterogeneities at the subnational level (i.e., between administration regions). For the study period of 2005-2019, we analyzed geographic and population factors for each of Senegal's 14 administrative regions to examine potential associations with DTP3 coverage. The analysis was conducted as part of the Vaccine Exemplars Program, the purpose of which

For peer review only - http://bmjopen.bmj.com/site/about/guidelines.xhtml

was to identify how and why some countries have achieved better-than-average and sustained coverage of early childhood vaccines [11, 19-21].

Data sources

Household-level immunization data was collected from Senegal's Demographic and Health Surveys (DHS) (years 2005, 2010/2011, 2012/2013, and yearly from 2014 to 2019) [22], with data provided for all 14 regions and all years (notably 3 of the regions were created/split from existing regions in 2008; coverage for these regions in 2005 is estimated to be equal to that of the original combined regions). The data can be accessed upon approval from the DHS Program [23]. DHS surveys were sampled to be representative at the regional level, and thus we were not able to analyze statistical associations at the smaller district level. 28 sampling strata are created which correspond to urban and rural parts for each of the 14 regions; samples are drawn independently from these sampling strata. Specific details on sampling methods for each survey year can be found in the appendices of each report (see [22]). Data extracted from the surveys include DTP3 vaccination (recommended administration to children at 14-weeks-old in Senegal), residence type (urban or rural), female literacy, proportion receiving prenatal care from skilled providers, and self-reported problems accessing care when sick (i.e., women were asked if distance, permission, money, and traveling alone were significant barriers when seeking treatment).

Child immunization is covered in the women's questionnaire in DHS surveys with responses representing each child of the mother surveyed. Literacy is assessed in the survey by the questioner asking the respondent to read from a card [24]. Prenatal (or antenatal) care is assessed by asking women who attended their last birth, with the following options: doctor, nurse/midwife, auxiliary nurse/midwife, community health worker, other health worker, traditional birth attendant, other, and no antenatal care.

Region-specific population density and poverty (reported for year 2018) were determined from Senegal national reports [25]. Poverty incidence is estimated using the cost of basic needs approach, which considers the proportion of the population unable to meet their basic needs and combines a country-

For peer review only - http://bmjopen.bmj.com/site/about/guidelines.xhtml

specific food poverty line and non-food poverty line. For more details with respect to this calculation specific to Senegal, see [26]. We collected regional counts of skilled healthcare workers from the Ministère de la Santé et de l'Action sociale (MSAS) (2018) [27].

Data analysis

Bivariate and multivariate logistic and linear regression analyses were performed to determine statistical associations of factors with respect to coverage of DTP3; factors included in multivariate analysis include residence type (urban or rural), female literacy, self-reported problems accessing care when sick, and prenatal care provider. We calculated DTP3 coverage – the dependent variable in our models - for each region of Senegal using DHS data for each year available during the study period. The immunization coverage for a region was calculated as the percentage of living children ages 12-23 months represented in the survey for that region who received DTP3. Data must be weighted because the overall probability of a household being selected is not constant across regions and residence types [28]. Thus, we compute the individual weight for women and households to adjust for differences in probability of selection and response between cases in a sample. We filter the data for living children ages 12-23 months. For the time series data, we do this for each year of data available. A vaccination is considered administered if it is reported by the mother or marked on the vaccination card with or without a date.

For our analysis, we consider a woman to be literate if she is able to read a whole sentence or part of a sentence as reported in the DHS survey [24, 28]. We consider a woman to have received skilled prenatal care if she answered "doctor", "nurse/midwife", or "auxiliary nurse/midwife" as attending her most recent birth.

We calculated the Pearson correlation coefficients (r) to determine statistically significant correlations between the quantitative factors and between factors and DTP3 coverage. We clustered regions geographically into 2 groups (coastal and inland), calculated the variance in DTP3 values over time for each region, and performed independent sample t-tests to compare these two groups of variances.

For peer review only - http://bmjopen.bmj.com/site/about/guidelines.xhtml

Patient and public involvement

There was no patient or public involvement in the study design of this research, the interpretation of the results, or the writing or editing of this document. All data used in this analysis was de-identified public health data.

Results

Table 1 reports the number of DHS survey respondents (unweighted) in each survey data set which fit the criteria for inclusion (i.e., with living children aged 12-23 months). The number of respondents in each region per year is reported in Table S1 in the supplemental material.

SURVEY YEAR	RESPONDENTS FITTING CRITERIA
**2005**	2138
**2010/2011**	2377
**2012/2013**	1329
**2014**	2662
**2015**	1310
**2016**	1323
**2017**	2390
**2018**	1337
**2019**	1183

Table 1 Senegal household survey data years and unweighted number of survey respondents meeting criteria for inclusion.

Factors associated with DTP3 coverage by administrative region

For each region in Senegal, Table S2 in the supplemental material reports the weighted percentage of survey respondents with "yes" responses per household factor for year 2019; data is filtered for respondents who meet inclusion criteria. DTP3 coverage by region ranged from 73% to 100% in 2019

For peer review only - http://bmjopen.bmj.com/site/about/guidelines.xhtml

(Figure 1, Table S2). Higher DTP3 coverage rates were observed in Dakar (the most populous region and includes the capital city) and its surrounding regions, and along the coast, as well as in the landlocked region of Matam. In 2018, there were approximately 6,600 people per km^2 in Dakar, which is predominantly urban (97% urban) [29]. The population density in other regions was at most 362 people per km^2. Population density does not have a statistically significant correlation with DTP3 coverage at the regional level, though this correlation is higher when excluding Dakar (Pearson's correlation r=0·39, p=0·19).

Unadjusted bivariate analyses (Figure 2) revealed significant associations (p=0·05) between higher DTP3 vaccination and the following:

- positive associations:
  - type of place of residence (urban) (β=0·61, p=0·0157),
  - higher female literacy (β=1·11, p=0·0007),
  - higher skilled prenatal care (β=1·80, p<0·0001), and
- negative associations:
  - more barriers when seeking treatment with respect to
    - distance (β=-0·70, p=0·0009) and
    - traveling alone (β=-1·08, p<0·0001).

We find that significant associations hold for female literacy, skilled prenatal care, distance, and traveling alone when adjusting by type of place of residence (urban).

Multivariate regression revealed correlations between factors (Figure 2), with only 3 factors remaining statistically significant: positive associations with female literacy (β=0·92, p=0·0065) and skilled prenatal care (β=1·58, p<0·0001), and negative associations with more barriers when seeking treatment with

For peer review only - http://bmjopen.bmj.com/site/about/guidelines.xhtml

respect to traveling alone (β=-0·82, p=0·0026) (more details provided in Table S3 in the supplemental materials). We also found correlations between population density and fewer barriers when seeking treatment with respect to distance (r=0·61, p=0·02) and traveling alone (r=0·48, p=0·08); traveling alone is correlated with distance (r=0·51, p<0·0001).

Coastal regions, namely, Dakar and Ziguinchor, have the highest female literacy rates (68% and 70%, respectively). The percentage of women who received skilled prenatal care from a doctor, nurse, or midwife was lowest in Tambacounda and Kedougou (88% and 85%, respectively), compared to over 95% in other regions (Figure 1).

Poverty level did not statistically explain regional differences in DTP3 coverage (bivariate regression coefficient β=-0·17, p=0·157), potentially due to heterogeneity within regions. We also found no statistical associations between DTP3 coverage and the number of doctors, nurses, and midwives per 100,000 population using bivariate or multivariate regression analysis (Figure 3, more details provided in Table S4 in the supplemental materials).

Access to Health Services

Women in regions with low DTP3 coverage reported more barriers to accessing care with respect to (i) traveling alone to seek care and (ii) distance from healthcare facilities. In the more remote, inland regions of Senegal, as many as 42% of female respondents in 2019 identified traveling alone to seek care as a significant barrier to accessing care. Distance to healthcare facilities was also a substantial barrier to accessing care in these regions of Senegal (identified as a barrier by 50% of female respondents in Tambacounda, the largest region by geographical area). Sociodemographic characteristics of this survey group are reported in Table S5 in the supplemental materials [30].

For peer review only - http://bmjopen.bmj.com/site/about/guidelines.xhtml

Coverage over time by administrative region and geographic location

DTP3 coverage from 2005 to 2019 by region in Senegal is reported in Figure 4 [31]. We observed that the DTP3 coverage was consistently higher for the west and southwestern regions located near Dakar and along the coast, and the variance of DTP3 coverage over time was consistently lower for this group of coastal regions (t-test, p=0.076) compared to the group of eastern and northern regions. The region with the lowest DTP3 coverage in 2019 was Kolda (73%), following a sharp drop in DTP3 coverage after 2017 compared to previous years. The rural and landlocked regions in the north and east demonstrated more variability in coverage over time. However, among these regions, Tambacounda and Kedougou saw large improvements, from 73% to 86% and 60% to 83%, respectively between 2017 and 2019; notably, Matam saw the largest increase (from 73% to 100%) in coverage among all regions between 2010 and 2019.

Discussion

We conducted a study to assess the demographic associations of early childhood vaccination within Senegal. Observing heterogeneities in DTP3 coverage across Senegal and comparing those differences with demographic and geographic factors may aid decision-makers in addressing disparities in vaccination access and uptake. Our analysis of household level data revealed associations between DTP3 coverage and 1) female literacy, 2) access to skilled prenatal care, and 3) challenges accessing care while traveling alone.

Early childhood vaccination in Senegal steadily increased for all recommended vaccines from 2002 to 2007, with coverage remaining mostly constant through 2019 [11, 32-37]. Based on an analysis of qualitative data including interviews with Senegalese administrators at various levels and focus group discussions with caregivers and community health workers, Senegal's immunization program has been successful due to "*strong governance, collaboration, evidence-based decision-making, community ownership, and an overall commitment to health and prioritization of vaccine programming from all*

For peer review only - http://bmjopen.bmj.com/site/about/guidelines.xhtml

stakeholders and government officials."[11] Understanding the existing disparities between regions in Senegal, alongside related factors, may support improvements in health equity through utilization of Senegal's existing governance structures and strong community health worker program.

*Female Literacy & Maternal Education***.** We found that higher female literacy was significantly associated with higher DTP3 coverage. Female literacy and higher maternal education have been shown to be positively associated with higher vaccination coverage globally [18, 38-40]; it is atypical that Senegal, with low female literacy at the national level [41], has relatively high DTP3 coverage compared to other LMICs. While mothers with high literacy and education may be more likely to independently seek vaccinations for their children [42], the health system in Senegal has been able to reach the more vulnerable rural communities with lower female literacy through community health workers and community-based health huts; "health huts are the backbone of Senegal's community health system, and they deserve much credit for Senegal's impressive progress on disease prevention and treatment, including its far-reaching immunization program" [9]. Involvement of community health workers such as respected female elders known as "bajenu gox" have served as a link between the health care services (i.e., health centers, health posts, health huts) and the communities, facilitating and coordinating immunization outreach activities [10].

*Access to Healthcare Workers & Facilities***.** We found that skilled prenatal care was associated with higher DTP3 coverage in Senegal, which aligns with existing literature. Access to skilled healthcare workers has an important role in childhood vaccination. Limited access to skilled prenatal care has been linked to under-vaccination, or children not receiving all recommended vaccines [15, 43], and a prior study highlighted an association between the number of skilled healthcare workers (nurses and midwives) and DTP3 coverage using a cross-country analysis of developing countries around the world for the years 1990 to 2004 [40]. Self-reported barriers limiting access to care for women when seeking treatment,

For peer review only - http://bmjopen.bmj.com/site/about/guidelines.xhtml

namely traveling alone to healthcare facilities and travel distance, were associated with DTP3 vaccination. In regions with higher population density, women report fewer concerns regarding traveling alone to access care when sick. Rural communities report more challenges than urban communities with respect to accessing immunization services. Unfortunately, retaining skilled health workers is more difficult in rural regions of Senegal [44]. Senegal has addressed these challenges through training community health workers and health post staff, improving infrastructure for outreach services, increasing capacity for health post expansion, and improving surveillance and data management at local levels [11]. In 2011, a 5-year $40 million grant from USAID helped Senegal construct, maintain, and link more health huts to the national health system [45].

Our results illustrate the role of contextual factors in relation to childhood vaccination. Although national childhood vaccination coverage in Senegal is relatively high compared to other LMICs, there are still gaps in coverage and disparities between regions which, similar to other studies, illustrate inequities in deployment of vaccination services [12, 13]. National health programs rely on the consideration of contextual factors and heterogeneity between subnational areas, such as those identified in this study, to inform programmatic decision-making and improve health equity. Strategies for improving immunization rates need to be tailored considering subnational differences; as stated in The Immunization Agenda 2030, "reaching all people will require higher national vaccination coverage, but also less subnational inequity" [46]. Subnational childhood vaccination has been negatively correlated with institutional mistrust [47]. Strong governance structures are required to prevent and address institutional mistrust while prioritizing equity of healthcare access at the national level [11, 12, 48]. Subnational DTP3 coverage estimates have been compared with national health spending, GDP per capita, and other governance indicators in sub-Saharan Africa to investigate the drivers of equitable immunization services [49]. To reduce differences in vaccination coverage between regions, and address institutional mistrust, national health policies and programs should consider heterogeneity between and within regions. Senegal has implemented top-down

For peer review only

For peer review only - http://bmjopen.bmj.com/site/about/guidelines.xhtml

and bottom-up mechanisms for decision-making, planning, and resource allocation to address subnational heterogeneity and allow for strategies and initiatives tailored for local populations [11].

Strengths and limitations

This work uses regionally representative data sets to explain associations between DTP3 coverage and demographic and health system characteristics at the subnational level within Senegal, and evaluates changes in vaccination coverage over time for each of these regions, considering the geographic location of each region. We focused on analyzing respondent groups at the regional level or higher because DHS surveys were not sampled to be representative at the smaller district level. Heterogeneities at the subregional level were thus not evaluated. If district level data were available, we conjecture that negative associations between DTP3 coverage and other factors, such as poverty, could be identified, given heterogeneities at the subregional level. For instances where DTP3 immunization was determined based on maternal recall, errors in recall could have biased results. Additionally, immunization is only reported in the women's questionnaire of the DHS survey; bias may exist due to families of children with no mothers.

Conclusions

Senegal has improved vaccination coverage across its 14 administrative regions despite high poverty and low female literacy as compared to other LICs and LMICs [19]. While coverage varies between regions, high coverage has expanded in recent years beyond coastal and urban regions into the inland and rural areas. National and regional efforts - especially increasing the number of health posts and huts to reach rural communities and expanding the community health worker programs - have likely played an important role [9]. Historically, the inland and rural regions have had variability in coverage over time; future years will tell if the recent increases in coverage will be sustained.

For peer review only - http://bmjopen.bmj.com/site/about/guidelines.xhtml

List of abbreviations

DAH: Development assistance for health

DHS: Demographic and Health Surveys

DTP3: Third dose of the diphtheria-tetanus-pertussis vaccine

Gavi: Gavi, The Vaccine Alliance

GVAP: Global Vaccine Action Plan

LIC: Low income country

LMIC: Lower-middle income country

MSAS: Ministère de la Santé et de l'Action sociale

WHO: World Health Organization

Declarations

Ethics approval and consent to participate

The current research was approved as exempt human research by the Institutional Review Board committee of Emory University, Atlanta, Georgia, USA (IRB00111474). All DHS Program procedures and questionnaires are reviewed and approved by ICF Institutional Review Boards. The authors of this paper received permission from the DHS Program for the use of the data.

All DHS survey respondents remained anonymous to the researchers.

Consent for publication

Not applicable

For peer review only

Availability of data and materials

The data that support the findings of this study are available at https://www.dhsprogram.com/data/available-datasets.cfm.

Competing interests

The authors declare that there are no competing interests.

Funding

Funding for this study was provided by the Bill & Melinda Gates Foundation, OPP1195041.

Authors' contributions

HS, FC, PK, and DN contributed to study structure. HS analyzed DHS and national data and HS, FC, PK, and DN synthesized key themes. ZS, MF, and MS provided essential context relating to qualitative factors within Senegal. All authors contributed to drafting the article and approve of the final manuscript.

Acknowledgements

The authors thank the referees for thoughtful and constructive comments which significantly improved this paper. This research was supported in part by the following Georgia Tech benefactors: William W. George, Andrea Laliberte, Joseph C. Mello, Richard "Rick" E. and Charlene Zalesky.

Figure legends

Figure 1 DTP3 coverage by region of Senegal and regional proportions of select metrics [30]. "Skilled Prenatal Care" refers to the proportion of women ages 15-49 who had a live birth within 5 years before the survey who had prenatal care provided by a skilled health care worker (2017). Skilled care includes doctor, nurse, and midwife. Unskilled care includes matron/traditional midwife, "other", or none. "Ease

For peer review only

of Access to Care – Traveling Alone”, refers to the proportion of female survey respondents by region who did not consider traveling alone to be a significant problem with accessing care when sick (2017).

Figure 2 Regression analysis for household-level factors associated with DTP3 vaccination by DHS 2019 survey response per living child aged 12-23 months (weighted N=1129). The bars correspond to 95% confidence intervals. Bars which do not intersect 0 correspond to statistical associations with DTP3 vaccination (< 0 implies negative association, > 0 implies positive association).

Figure 3 Regression analysis for region-level factor associations with DTP3 vaccination. Poverty incidence is estimated using the cost of basic needs approach, which considers the proportion of the population unable to meet their basic needs and combines a country-specific food poverty line and non-food poverty line [26]. The bars correspond to 95% confidence intervals.

Figure 4 DTP3 coverage in Senegal by year and region (DHS 2005-2019). The regions of Kaffrine, Sedhiou, and Kedougou were split from existing regions in 2008 [31].

REFERENCES

1. Rémy V, Zöllner Y, Heckmann U. Vaccination: the cornerstone of an efficient healthcare system. J Mark Access Health Policy. 2015;3:10.3402/jmahp.v3.27041.
2. Ozawa S, Clark S, Portnoy A, Grewal S, Brenzel L, Walker DG. Return On Investment From Childhood Immunization In Low- And Middle-Income Countries, 2011-20. Health Aff (Millwood). 2016;35(2):199-207.
3. World Health Organization. Immunization coverage 2020 2021 [Available from: https://www.who.int/news-room/fact-sheets/detail/immunization-coverage.
4. World Health Organization. Global vaccine action plan 2011-2020 2013 [Available from: https://www.who.int/publications/i/item/global-vaccine-action-plan-2011-2020.
5. World Health Organization. 2018 assessment report of the Global Vaccine Action Plan: strategic advisory group of experts on immunization 2018 [Available from: https://apps.who.int/iris/handle/10665/276967.
6. World Health Organization. WHO vaccine-preventable diseases: monitoring system. 2020 global summary. WHO UNICEF estimates time series for Senegal (SEN) 2020 [Available from: https://apps.who.int/immunization_monitoring/globalsummary/estimates?c=SEN.
7. IHME. Global health spending 1995-2016 2019 [Available from: http://ghdx.healthdata.org/record/ihme-data/global-health-spending-1995-2016

For peer review only - http://bmjopen.bmj.com/site/about/guidelines.xhtml

8. WHO and UNICEF. WHO-UNICEF joint reporting form: immunization financing indicators, 2019 [Available from: https://www.who.int/immunization/programmes_systems/financing/data_indicators/en/.
9. Exemplars in Global Health. What did Senegal do? 2021 [Available from: https://www.exemplars.health/topics/under-five-mortality/senegal/what-did-senegal-do.
10. Devlin K, Pandit-Rajani T, Egan KF. Senegal's Community-based Health System Model: Structure, Strategies, and Learning. Arlington, VA: Advancing Partners & Communities; 2019.
11. Sakas Z, Hester KA, Rodriguez K, Diatta SA, Ellis AS, Gueye DM, et al. Critical success factors for high routine immunization performance: A case study of Senegal. Vaccine: X. 2023;14:100296.
12. Faye CM, Wehrmeister FC, Melesse DY, Mutua MKK, Maïga A, Taylor CM, et al. Large and persistent subnational inequalities in reproductive, maternal, newborn and child health intervention coverage in sub-Saharan Africa. BMJ Global Health. 2020;5(1):e002232.
13. Mosser JF, Gagne-Maynard W, Rao PC, Osgood-Zimmerman A, Fullman N, Graetz N, et al. Mapping diphtheria-pertussis-tetanus vaccine coverage in Africa, 2000–2016: a spatial and temporal modelling study. The Lancet. 2019;393(10183):1843-55.
14. Utazi CE, Thorley J, Alegana VA, Ferrari MJ, Takahashi S, Metcalf CJE, et al. High resolution age-structured mapping of childhood vaccination coverage in low and middle income countries. Vaccine. 2018;36(12):1583-91.
15. Eze P, Agu UJ, Aniebo CL, Agu SA, Lawani LO, Acharya Y. Factors associated with incomplete immunisation in children aged 12–23 months at subnational level, Nigeria: a cross-sectional study. BMJ Open. 2021;11(6):e047445.
16. Sarker AR, Akram R, Ali N, Chowdhury ZI, Sultana M. Coverage and Determinants of Full Immunization: Vaccination Coverage among Senegalese Children. Medicina. 2019;55(8).
17. Ameyaw EK, Kareem YO, Ahinkorah BO, Seidu A-A, Yaya S. Decomposing the rural–urban gap in factors associated with childhood immunisation in sub-Saharan Africa: evidence from surveys in 23 countries. BMJ Global Health. 2021;6(1):e003773.
18. Mbengue MAS, Sarr M, Faye A, Badiane O, Camara FBN, Mboup S, et al. Determinants of complete immunization among senegalese children aged 12-23 months: evidence from the demographic and health survey. BMC Public Health. 2017;17(1):630.
19. Bednarczyk RA, Hester KA, Dixit SM, Ellis AS, Escoffery C, Kilembe W, et al. Exemplars in vaccine delivery protocol: a case-study-based identification and evaluation of critical factors in achieving high and sustained childhood immunisation coverage in selected low-income and lower-middle-income countries. BMJ Open. 2022;12(4):e058321.
20. Hester KA, Sakas Z, Ellis AS, Bose AS, Darwar R, Gautam J, et al. Critical success factors for high routine immunization performance: A case study of Nepal. Vaccine: X. 2022;12:100214.
21. Micek K, Hester KA, Chanda C, Darwar R, Dounebaine B, Ellis AS, et al. Critical success factors for routine immunization performance: A case study of Zambia 2000 to 2018. Vaccine: X. 2022;11:100166.
22. The DHS Program. Senegal Continuous Demographic Health Survey 2005-2019 2019 [Available from: https://www.dhsprogram.com/data/available-datasets.cfm.
23. DHS Program. Demographic and Health Surveys: Data 2023 [Available from: https://www.dhsprogram.com/Data/.
24. The DHS Program. New Data Available from DHS-7 Questionnaire: Literacy, Ownership of Goods, Internet Use, Finances, and Tobacco Use 2017 [Available from: https://blog.dhsprogram.com/dhs7-part-3/.
25. Knoema. Senegal 2021 [Available from: https://knoema.com/atlas/Senegal.
26. Agence Nationale de la Statistique et de la Démographie (ANSD). Deuxieme Enquete De Suivi De La Pauvrete Au Senegal (ESPS-11 2011) 2013 [Available from: https://www.ansd.sn/ressources/publications/Rapport_ESPS-2011.pdf.

For peer review only - http://bmjopen.bmj.com/site/about/guidelines.xhtml

27. Ministère de la Santé et de l'Action sociale. Annuaire des statistiques sanitaires et sociales 2018 2020 [Available from: https://sante.sec.gouv.sn/sites/default/files/Annuaire%20Satatistiques%20sanitaires%20et%20sociales%202018.pdf.

28. Croft TN, Marshall AMJ, Allen CK, et al. Guide to DHS Statistics. Rockville, Maryland, USA: ICF.2018.

29. Senegal Data Portal. Population & Financial Statistics of Senegal, 2011 2012 [Available from: https://senegal.opendataforafrica.org/SEPFS2016/population-financial-statistics-of-senegal-2011.

30. Agence Nationale de la Statistique et de la Démographie (ANSD). Sénégal : Enquête Démographique et de Santé Continue (EDS-Continue) 2019 2020 [Available from: https://dhsprogram.com/pubs/pdf/FR368/FR368.T.pdf.

31. Monkam NF. Property Taxation in Senegal: Legislation and Practice. Journal of Property Tax Assessment & Administration. 2011;8(3):41-60.

32. WHO and UNICEF. Senegal: WHO and UNICEF estimates of immunization coverage: 2020 revision 2021 [Available from: https://cdn.who.int/media/docs/default-source/country-profiles/immunization/immunization_sen_2021.pdf.

33. Unicef. Immunization data July 2021 2021 [Available from: https://data.unicef.org/resources/dataset/immunization/.

34. Russo G, Lihui X, McIsaac M, Matsika-Claquin MD, Dhillon I, McPake B, et al. Health workers' strikes in low-income countries: the available evidence. Bulletin of the World Health Organization. 2019.

35. HRH2030 Program. Three Questions with Dr. Matar Camara: A public health specialist with a vision 2018 [Available from: https://hrh2030program.org/3-questions-with-matar-camara-2/.

36. Despite Senegal's Nine-Month Health Worker Strike, Nioro Sees LARC Usage Trend Improve: TCI: The Challenge Initiative; 2020 [Available from: https://tciurbanhealth.org/despite-senegals-nine-month-health-worker-strike-nioro-sees-positive-impact-from-larcs/.

37. WHO and UNICEF. Senegal: WHO and UNICEF estimates of immunization coverage: 2019 revision 2020 [Available from: https://www.who.int/immunization/monitoring_surveillance/data/sen.pdf.

38. Streatfield K, Singarimbun M, Diamond I. Maternal Education and Child Immunization. Demography. 1990;27(3):447-55.

39. Vikram K, Vanneman R, Desai S. Linkages between maternal education and childhood immunization in India. Soc Sci Med. 2012;75(2):331-9.

40. Anand S, Bärnighausen T. Health workers and vaccination coverage in developing countries: an econometric analysis. The Lancet. 2007;369(9569):1277-85.

41. The World Bank. Literacy rate, adult female 2020 [Available from: https://data.worldbank.org/indicator/SE.ADT.LITR.FE.ZS

42. Balogun SA, Yusuff HA, Yusuf KQ, Al-Shenqiti AM, Balogun MT, Tettey P. Maternal education and child immunization: the mediating roles of maternal literacy and socioeconomic status. (1937-8688 (Electronic)).

43. Rainey JJ, Watkins M, Ryman TK, Sandhu P, Bo A, Banerjee K. Reasons related to non-vaccination and under-vaccination of children in low and middle income countries: Findings from a systematic review of the published literature, 1999–2009. Vaccine. 2011;29(46):8215-21.

44. Zurn P, Codjia L Fau - Sall FL, Sall Fl Fau - Braichet J-M, Braichet JM. How to recruit and retain health workers in underserved areas: the Senegalese experience. Bull World Health Organ.88(5):386-9.

45. ChildFund. Senegal's Commitment to Community Health Yields Benefits 2021 [Available from: https://www.childfund.org/senegals-commitment-to-community-health/.

46. World Health Organization. Immunization Agenda 2030: A Global Strategy to Leave No One Behind. World Health Organization; 2020.

For peer review only

For peer review only - http://bmjopen.bmj.com/site/about/guidelines.xhtml

47. Stoop N, Hirvonen K, Maystadt J-F. Institutional mistrust and child vaccination coverage in Africa. BMJ Global Health. 2021;6(4):e004595.
48. Shen AK, Fields R, McQuestion M. The future of routine immunization in the developing world: challenges and opportunities. Glob Health Sci Pract. 2014;2(4):381-94.
49. Ikilezi G, Augusto OJ, Sbarra A, Sherr K, Dieleman JL, Lim SS. Determinants of geographical inequalities for DTP3 vaccine coverage in sub-Saharan Africa. Vaccine. 2020;38(18):3447-54.

For peer review only

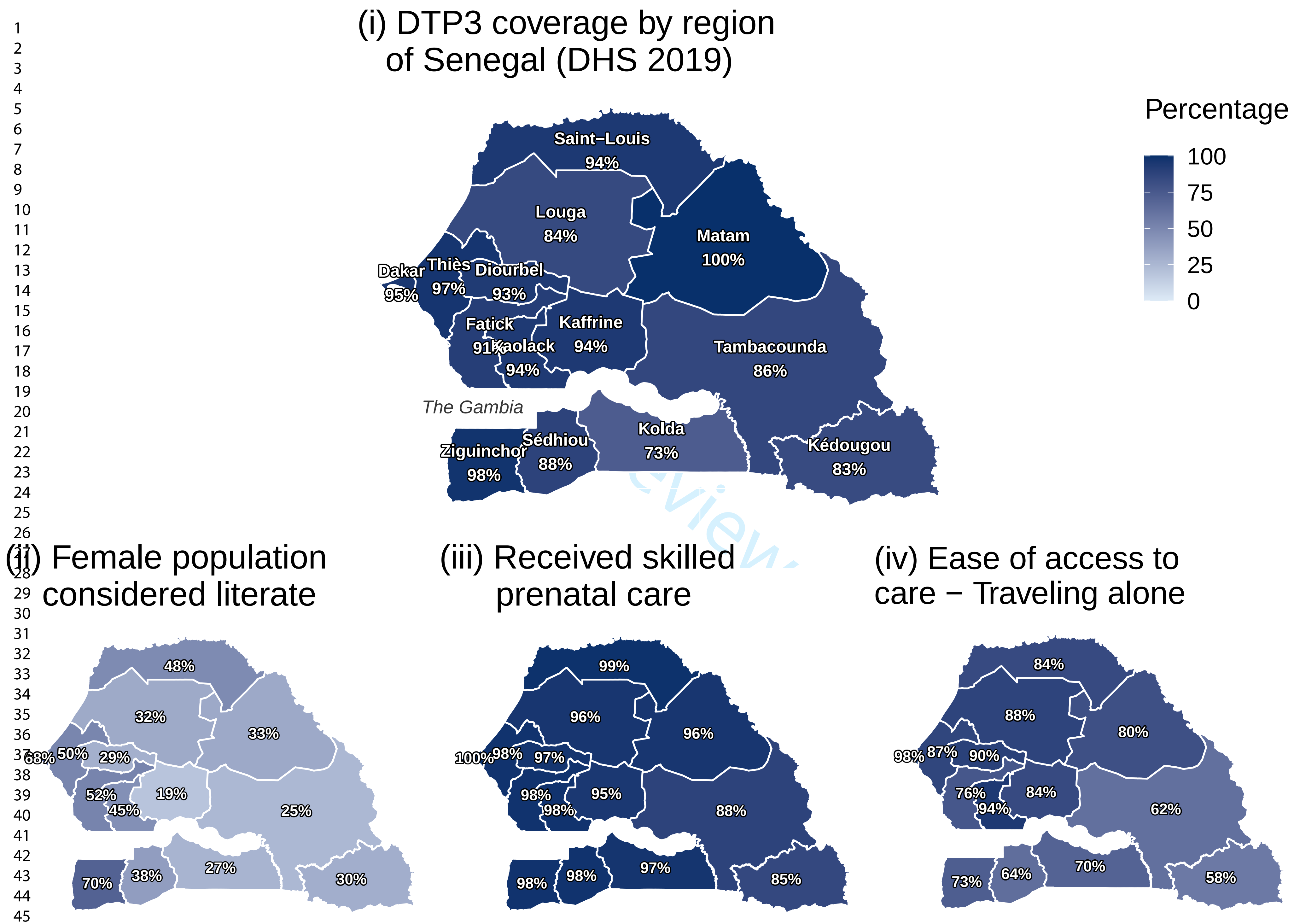
(i) DTP3 coverage by region of Senegal (DHS 2019)
Percentage
100
75
50
25
0
Saint−Louis 94%
Louga 84%
Matam 100%
Dakar 95%
Thiès 97%
Diourbel 93%
Fatick 91%
Kaffrine 94%
Kaolack 94%
Tambacounda 86%
The Gambia
Kolda 73%
Sédhiou 88%
Ziguinchor 98%
Kédougou 83%
(ii) Female population considered literate
48%
32%
33%
68%
50%
29%
52%
19%
45%
25%
70%
38%
27%
30%
(iii) Received skilled prenatal care
99%
96%
96%
100%
98%
97%
98%
95%
98%
88%
98%
98%
97%
85%
(iv) Ease of access to care − Traveling alone
84%
88%
80%
98%
87%
90%
76%
84%
94%
62%
73%
64%
70%
58%

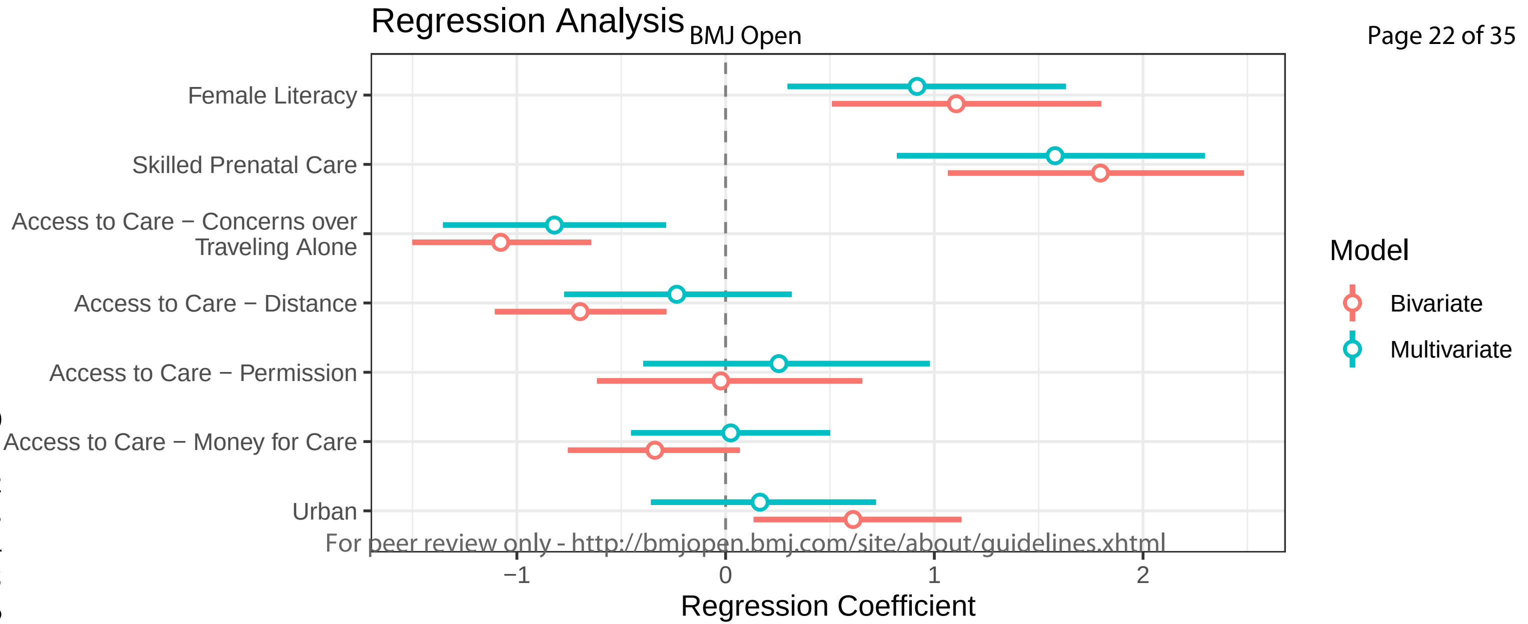

For peer review only - http://bmjopen.bmj.com/site/about/guidelines.xhtml

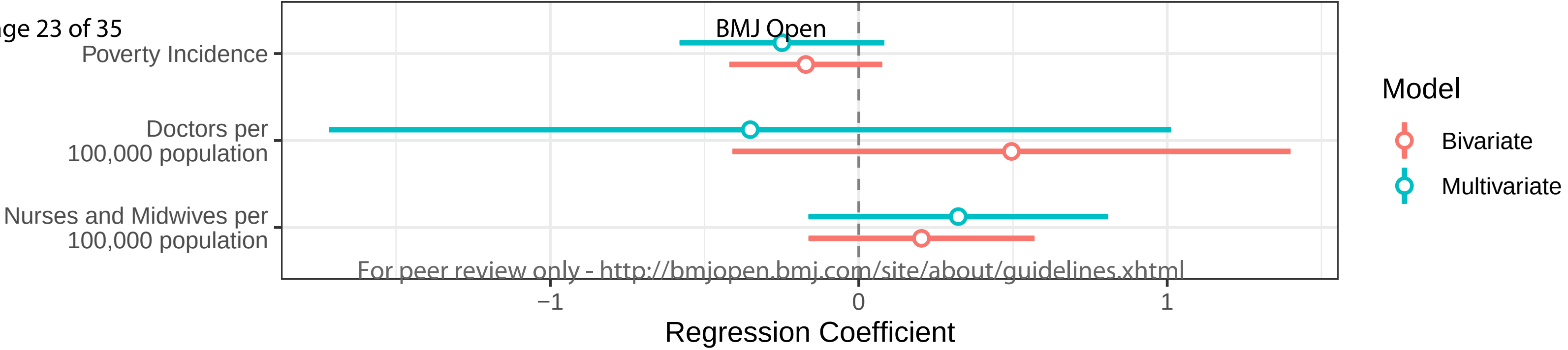

For peer review only - http://bmjopen.bmj.com/site/about/guidelines.xhtml

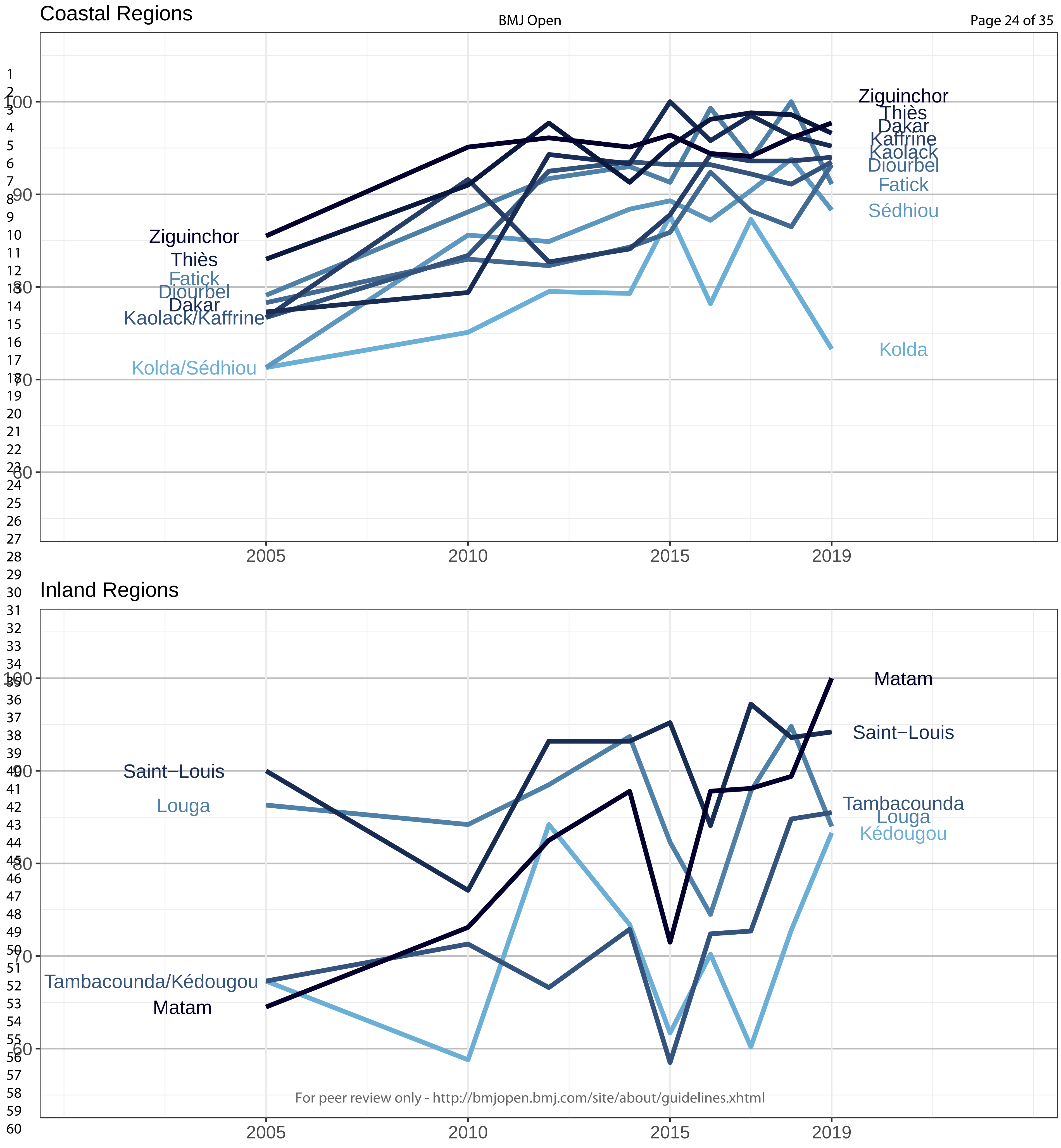
Coastal Regions
Ziguinchor
Thiès
Fatick
Diourbel
Dakar
Kaolack/Kaffrine
Kolda/Sédhiou
Ziguinchor
Thiès
Dakar
Kaffrine
Kaolack
Diourbel
Fatick
Sédhiou
Kolda
100
90
80
70
60
2005
2010
2015
2019
Inland Regions
Saint−Louis
Louga
Tambacounda/Kédougou
Matam
Matam
Saint−Louis
Tambacounda
Louga
Kédougou
100
90
80
70
60
2005
2010
2015
2019

REGION	2005	2010/2011	2012/2013	2014	2015	2016	2017	2018	2019
Dakar	150	141	70	130	52	63	164	80	60
Diourbel	229	206	139	259	113	107	182	110	107
Fatick	178	185	77	163	94	97	184	90	63
Kaffrine	*	188	118	245	116	140	217	161	106
Kaolack	255	217	131	259	146	124	156	119	130
Kedougou	*	87	66	134	78	100	142	76	60
Kolda	232	190	108	192	132	104	164	94	93
Louga	198	165	82	199	97	86	158	92	77
Matam	178	160	92	182	87	96	180	84	94
Saint-Louis	169	157	87	179	59	93	137	79	67
Sedhiou	*	197	95	175	83	78	196	92	85
Tambacounda	183	178	99	222	91	103	201	106	91
Thies	232	179	118	214	111	95	190	98	107
Ziguinchor	134	127	47	109	51	37	119	56	43
TOTAL	2138	2377	1329	2662	1310	1323	2390	1337	1183

***** *Kaffrine, Kedougou, and Sedhiou were split from existing regions in 2008.*

Table S1 Unweighted number of survey respondents fitting survey criteria (living children ages 12-24 months) by year and region.

For peer review only - http://bmjopen.bmj.com/site/about/guidelines.xhtml

Region	DTP3	Female Literacy	Skilled Prenatal Care	Access to Care Concerns				Urban
				Traveling Alone	*Distance*	*Permission*	*Money*	
**Dakar**	95.2%	45.2%	100.0%	12.1%	14.8%	2.9%	36.0%	95.2%
**Diourbel**	93.2%	14.3%	99.3%	2.8%	30.0%	9.2%	52.7%	9.7%
**Fatick**	91.1%	33.2%	96.4%	12.4%	35.2%	8.9%	45.4%	24.8%
**Kaffrine**	94.0%	13.1%	98.2%	19.2%	21.4%	25.5%	46.0%	13.8%
**Kaolack**	93.5%	24.8%	98.4%	3.7%	34.7%	1.3%	53.4%	31.2%
**Kedougou**	83.3%	24.6%	89.6%	21.0%	7.0%	19.9%	32.3%	20.2%
**Kolda**	73.3%	23.7%	93.5%	45.2%	44.5%	2.0%	61.3%	20.7%
**Louga**	84.0%	26.3%	95.9%	26.5%	40.8%	18.6%	60.4%	17.9%
**Matam**	100.0%	10.3%	97.4%	20.5%	30.9%	21.3%	84.3%	13.8%
**Saint-Louis**	94.2%	27.8%	97.0%	13.8%	40.0%	20.4%	70.5%	38.8%
**Sedhiou**	88.3%	28.5%	95.4%	50.2%	60.2%	2.4%	66.7%	12.6%
**Tambacounda**	85.5%	10.3%	91.8%	21.8%	2.9%	20.9%	39.3%	34.7%
**Thies**	96.6%	30.0%	100.0%	16.3%	27.7%	4.9%	45.7%	47.4%
**Ziguinchor**	97.7%	61.9%	97.2%	14.1%	16.6%	2.3%	43.6%	40.1%
**All Senegal**	92.1%	26.9%	97.8%	15.9%	28.2%	9.5%	50.9%	37.9%

Table S2 Weighted percentage of survey respondents with "yes" responses per household factor for year 2019. Data is filtered for respondents who meet inclusion criteria.

For peer review only - http://bmjopen.bmj.com/site/about/guidelines.xhtml

Variable	β Coefficients and 95% CI	p-value
Urban	0·16 (-0·36, 0·72)	0·547
**Female Literacy**	**0·92 (0·30, 1·63)**	**0·0065**
**Skilled Prenatal Care from a doctor, nurse, or midwife**	**1·58 (0·82, 2·30)**	**< 0·0001**
Problems experienced among respondents who sought care when sick: Women ages 15-49 were asked if the following were significant barriers when seeking treatment when they were sick.		
Access to Care - Distance	-0·23 (-0·77, 0·32)	0·400
Access to Care - Permission	0·26 (-0·39, 0·98)	0·463
**Access to Care - Concerns over Traveling Alone**	**-0·82 (-1·35, -0·28)**	**0·0026**
Access to Care - Money for Care	0·02 (-0·45, 0·50)	0·919

Table S3 Household-level factors associated with DTP3 vaccination by survey response per living child age 12-23 months (N=1129 (weighted)): multivariate regression analysis. DHS 2019.

For peer review only

Variable	Coefficients and 95% CI	p-value
Poverty Incidence	-0·249 (-0·58, 0·08)	0·126
Doctors per 100,000 population	-0·352 (-1·72, 1·01)	0·579
Nurses and Midwives per 100,000 population	0·323 (-0·16, 0·81)	0·170

Table S4 Region-level factor associations with DTP3 coverage at the regional level (N=14): multivariate linear regression analysis. DHS 2019.

For peer review only

For peer review only - http://bmjopen.bmj.com/site/about/guidelines.xhtml

Sociodemographic Characteristics of Survey Respondents (n=8,649)	
**Religion**	
Muslim	97%
Christian	3%
**Ethnicity**	
Wolof	40%
Poular	28%
Serer	15%
Mandingue/Soce	5%
Diola	4%
Soninke	3%
Other/non-Senegalese	5%
**Education**	
Primary	21%
Secondary or more	32%
**Marital Status**	
Single	30%
Married	65%
Divorced/separated	4%
Widow	1%
**Age**	
15-19	22%
20-24	19%
25-29	16%
30-34	15%
35-39	13%
40-44	8%
45-49	7%

Table S5 Sociodemographic characteristics of female survey respondents, 2019.

For peer review only

For peer review only - http://bmjopen.bmj.com/site/about/guidelines.xhtml

The RECORD statement – checklist of items, extended from the STROBE statement, that should be reported in observational studies using routinely collected health data.

	Item No.	STROBE items	Location in manuscript where items are reported	RECORD items	Location in manuscript where items are reported
**Title and abstract**					
	1	(a) Indicate the study's design with a commonly used term in the title or the abstract (b) Provide in the abstract an informative and balanced summary of what was done and what was found	(a) Abstract: Methods<br>(b) Abstract: Methods and Results<br>(page 2)	RECORD 1.1: The type of data used should be specified in the title or abstract. When possible, the name of the databases used should be included.<br>RECORD 1.2: If applicable, the geographic region and timeframe within which the study took place should be reported in the title or abstract.<br>RECORD 1.3: If linkage between databases was conducted for the study, this should be clearly stated in the title or abstract.	Abstract: Methods (page 2)
**Introduction**					
Background rationale	2	Explain the scientific background and rationale for the investigation being reported	Page 5		
Objectives	3	State specific objectives, including any prespecified hypotheses	Page 5		
**Methods**					
Study Design	4	Present key elements of study design early in the paper	Page 5-6		
Setting	5	Describe the setting, locations, and relevant dates, including periods of recruitment, exposure, follow-up, and data collection	Page 6		

For peer review only

Participants	6	*(a) Cohort study* - Give the eligibility criteria, and the sources and methods of selection of participants. Describe methods of follow-up<br>*Case-control study* - Give the eligibility criteria, and the sources and methods of case ascertainment and control selection. Give the rationale for the choice of cases and controls<br>*Cross-sectional study* - Give the eligibility criteria, and the sources and methods of selection of participants<br>*(b) Cohort study* - For matched studies, give matching criteria and number of exposed and unexposed<br>*Case-control study* - For matched studies, give matching criteria and the number of controls per case	Data analysis – page 7. Retrospective analysis – living children aged 12-23 months from DHS reports for each year.	RECORD 6.1: The methods of study population selection (such as codes or algorithms used to identify subjects) should be listed in detail. If this is not possible, an explanation should be provided.<br>RECORD 6.2: Any validation studies of the codes or algorithms used to select the population should be referenced. If validation was conducted for this study and not published elsewhere, detailed methods and results should be provided.<br>RECORD 6.3: If the study involved linkage of databases, consider use of a flow diagram or other graphical display to demonstrate the data linkage process, including the number of individuals with linked data at each stage.	Data analysis – page 7. Retrospective analysis – living children aged 12-23 months from DHS reports for each year.
Variables	7	Clearly define all outcomes, exposures, predictors, potential confounders, and effect modifiers. Give diagnostic criteria, if applicable.	Data sources and data analysis, pages 6-7	RECORD 7.1: A complete list of codes and algorithms used to classify exposures, outcomes, confounders, and effect modifiers should be provided. If these cannot be reported, an explanation should be provided.	Data sources and data analysis, pages 6-7
Data sources/ measurement	8	For each variable of interest, give sources of data and details of methods of assessment (measurement).	Data sources and data analysis, pages 6-7		

For peer review only

		Describe comparability of assessment methods if there is more than one group			
Bias	9	Describe any efforts to address potential sources of bias	Data analysis, page 7		
Study size	10	Explain how the study size was arrived at	All available DHS data for Senegal; data sources page 6		
Quantitative variables	11	Explain how quantitative variables were handled in the analyses. If applicable, describe which groupings were chosen, and why	Data sources and data analysis, page 6-7		
Statistical methods	12	(a) Describe all statistical methods, including those used to control for confounding<br>(b) Describe any methods used to examine subgroups and interactions<br>(c) Explain how missing data were addressed<br>(d) *Cohort study* - If applicable, explain how loss to follow-up was addressed<br>*Case-control study* - If applicable, explain how matching of cases and controls was addressed<br>*Cross-sectional study* - If applicable, describe analytical methods taking account of sampling strategy<br>(e) Describe any sensitivity analyses	Data analysis, page 7		

For peer review only

Data access and cleaning methods		…		RECORD 12.1: Authors should describe the extent to which the investigators had access to the database population used to create the study population.<br>RECORD 12.2: Authors should provide information on the data cleaning methods used in the study.	Retrospective analysis of DHS data
Linkage		…		RECORD 12.3: State whether the study included person-level, institutional-level, or other data linkage across two or more databases. The methods of linkage and methods of linkage quality evaluation should be provided.	Retrospective analysis of DHS data
**Results**					
Participants	13	(a) Report the numbers of individuals at each stage of the study (*e.g.*, numbers potentially eligible, examined for eligibility, confirmed eligible, included in the study, completing follow-up, and analysed)<br>(b) Give reasons for non-participation at each stage.<br>(c) Consider use of a flow diagram	Figure 2 caption reports included 2019 survey respondents. 14 regions considered in region-level analysis and stated throughout.	RECORD 13.1: Describe in detail the selection of the persons included in the study (*i.e.,* study population selection) including filtering based on data quality, data availability and linkage. The selection of included persons can be described in the text and/or by means of the study flow diagram.	Data analysis – page 7. Retrospective analysis – living children aged 12-23 months from DHS reports for each year.
Descriptive data	14	(a) Give characteristics of study participants (*e.g.*, demographic, clinical, social) and information on exposures and potential confounders<br>(b) Indicate the number of participants with missing data for each variable of interest	Supplemental materials Table 3		

For peer review only

		(c) *Cohort study* - summarise follow-up time (*e.g.*, average and total amount)			
Outcome data	15	*Cohort study* - Report numbers of outcome events or summary measures over time<br>*Case-control study* - Report numbers in each exposure category, or summary measures of exposure<br>*Cross-sectional study* - Report numbers of outcome events or summary measures	Throughout results		
Main results	16	(a) Give unadjusted estimates and, if applicable, confounder-adjusted estimates and their precision (e.g., 95% confidence interval). Make clear which confounders were adjusted for and why they were included<br>(b) Report category boundaries when continuous variables were categorized<br>(c) If relevant, consider translating estimates of relative risk into absolute risk for a meaningful time period	In main body results and supplemental materials Tables 1 and 2.		
Other analyses	17	Report other analyses done—e.g., analyses of subgroups and interactions, and sensitivity analyses	In main body results.		
**Discussion**					
Key results	18	Summarise key results with reference to study objectives	Beginning of Discussion page 13		

For peer review only

Limitations	19	Discuss limitations of the study, taking into account sources of potential bias or imprecision. Discuss both direction and magnitude of any potential bias	End of page 14	RECORD 19.1: Discuss the implications of using data that were not created or collected to answer the specific research question(s). Include discussion of misclassification bias, unmeasured confounding, missing data, and changing eligibility over time, as they pertain to the study being reported.	End of page 14
Interpretation	20	Give a cautious overall interpretation of results considering objectives, limitations, multiplicity of analyses, results from similar studies, and other relevant evidence	Throughout discussion		
Generalisability	21	Discuss the generalisability (external validity) of the study results	Discussion page 15		
**Other Information**					
Funding	22	Give the source of funding and the role of the funders for the present study and, if applicable, for the original study on which the present article is based	Page 17		
Accessibility of protocol, raw data, and programming code		..		RECORD 22.1: Authors should provide information on how to access any supplemental information such as the study protocol, raw data, or programming code.	Page 17

*Reference: Benchimol EI, Smeeth L, Guttmann A, Harron K, Moher D, Petersen I, Sørensen HT, von Elm E, Langan SM, the RECORD Working Committee. The REporting of studies Conducted using Observational Routinely-collected health Data (RECORD) Statement. *PLoS Medicine* 2015; in press.

For peer review only

*Checklist is protected under Creative Commons Attribution (CC BY) license.

For peer review only
